# Digestibility and Retention Time of Coastal Bermudagrass (*Cynodon dactylon*) Hay by Horses

**DOI:** 10.3390/ani9121148

**Published:** 2019-12-14

**Authors:** Tayler L. Hansen, Elisabeth L. Chizek, Olivia K. Zugay, Jessica M. Miller, Jill M. Bobel, Jessie W. Chouinard, Angie M. Adkin, Leigh Ann Skurupey, Lori K. Warren

**Affiliations:** Department of Animal Sciences, University of Florida, Gainesville, FL 32611, USA; tlhansen@cornell.edu (T.L.H.); creeksidefarm14@gmail.com (E.L.C.); ozugay@ufl.edu (O.K.Z.); horsewhisperer1230@hotmail.com (J.M.M.); jbrides2@ufl.edu (J.M.B.); jessie23@ufl.edu (J.W.C.); aadkin@ufl.edu (A.M.A.); leighann.skurupey@ndsu.edu (L.A.S.)

**Keywords:** alfalfa, equine, fiber, forage maturity, mathematical modeling, mean retention time, orchardgrass, rate of passage, warm-season grass

## Abstract

**Simple Summary:**

Longer retention of forages with increased fiber concentrations may be a compensatory digestive strategy in horses. We investigated the digestive characteristics of bermudagrass hay, a prominent warm-season grass in the southeast United States that has greater fiber concentrations than other common forages fed to horses. The morphological structure and photosynthetic pathway of warm-season grasses differ from cool-season grasses and legumes which may have important impacts on equine digestion and digesta transit through the gastrointestinal tract. The retention time of Coastal bermudagrass was longer than alfalfa or orchardgrass hay. The digestibility of Coastal bermudagrass decreased with increasing maturity, but the fiber digestibility of alfalfa and orchardgrass was similar to the earliest maturity of Coastal bermudagrass hay. The chemical composition of the plant cell wall influences diet digestibility and is a major difference between warm-season and cool-season forages. The increased retention time of Coastal bermudagrass allows for microbial fermentation to occur longer, adapting to more difficult-to-digest plant cell walls in warm-season forages. The decrease in diet digestibility when horses consume warm-season forages can be reduced by feeding early maturity forage, by harvesting hay at an earlier stage of growth or managing pastures in a vegetative state.

**Abstract:**

Bermudagrass (*Cynodon dactylon*) and other warm-season grasses are known for their increased fiber concentrations and reduced digestibility relative to cool-season grasses and legumes. This study investigated the digestive characteristics and passage kinetics of three maturities of Coastal bermudagrass hay. A 5 × 5 Latin square design experiment was used to compare the digestion of five hays: alfalfa (*Medicago sativa*, ALF), orchardgrass (*Dactylis glomerata*, ORCH), and Coastal bermudagrass harvested at 4 (CB 4), 6 (CB 6), and 8 weeks of regrowth (CB 8). Horses were fed cobalt-ethylenediaminetetraacetic acid (Co-EDTA) and ytterbium (Yb) labeled neutral detergent fiber (NDF) before an 84-h total fecal collection to determine digesta retention time. Dry matter digestibility was greatest for ALF (62.1%) and least for CB 6 (36.0%) and CB 8 diets (36.8%, SEM = 2.1; *p* < 0.05). Mean retention time was longer (*p* < 0.05) for Coastal bermudagrass (particulate 31.3 h, liquid 25.3 h) compared with ORCH and ALF (28.0 h, SEM = 0.88 h; 20.7 h, SEM = 0.70 h). Further evaluation of digesta passage kinetics through mathematical modeling indicated ALF had distinct parameters compared to the other diets. Differences in digestive variables between forage types are likely a consequence of fiber physiochemical properties, warranting further investigation on forage fiber and digestive health.

## 1. Introduction

Bermudagrass (*Cynodon dactylon*) is one of the most prominent forages in the southeast United States; however, some horse owners and equine professionals assume that bermudagrass, particularly the Coastal variety, is a lower quality hay due to increased fiber concentrations. Furthermore, feeding Coastal bermudagrass hay in this region has been implicated as a cause of ileocecal impaction in horses [1]. The increased fiber concentrations of Coastal bermudagrass and fine, soft texture have been hypothesized to contribute to impaction [2], but greater fiber concentration is a common characteristic among warm-season grasses. Bermudagrass and other grasses common to subtropical and tropical climates (e.g., bahiagrass, millet, sorghum) possess a series of anatomical and biochemical modifications for C4 photosynthesis that distinguish them from C3 plants. The Kranz anatomy of C4 plants features tightly bundled mesophyll cells that form a ring around bundle-sheath cells. The proximity of mesophyll and bundle-sheath cells allows for carbon concentrating mechanisms in photosynthesis, reducing photorespiration in C4 plants. Plants using C4 carbon fixation are more efficient than C3 carbon fixation in areas of drought, high temperatures, and low nutrient inputs [3]. However, C4 plants tend to a have lower nutritive value via greater fiber concentrations that can lead to decreased animal performance [4].

Greater forage fiber concentrations have long been associated with decreased diet digestibility [5]. Forage digestibility by horses decreases by half a percentage unit for every one percentage unit increase in NDF concentration [6]. Using equine fecal inoculum, Lowman et al. [7] reported that time to reach total gas production took longer for oat (*Avena sativa*) straw and wheat (*Triticum aestivum*) straw compared with alfalfa (*Medicago sativa*) hay and grass haylage. Furthermore, the specific type of dietary fiber (insoluble vs. soluble) alters in vitro digestibility measurements [8]. Not only fiber concentration, but the specific composition of hemicellulose, cellulose, and lignin in the plant cell may alter digestion by horses.

The degradation of forage fiber in the equine gastrointestinal tract may be influenced by digesta rate of passage (ROP); however, a consistent relationship between fiber concentration and digesta mean retention time (MRT) has not been shown in horses. Low nutritional value forages have a longer retention time than high-quality legumes [9], but no difference in MRT was observed when horses were fed similar forage species differing in fiber concentration [10,11]. The influence of fiber concentration on digesta MRT may be confounded by factors such as the level of intake and feed particle size [12]. Furthermore, low-fermentable dietary fibers alter ROP through changes in digesta viscosity in the small intestine [13]. Such changes may not be detectable in total tract mean retention time (TTMRT) calculations.

Several mathematical models have been used to describe digesta passage in ruminants that improve understanding of passage kinetics by estimating retention time in the rumen from fecal marker excretion [14,15,16]. These models have been applied to equine fecal marker excretion with the hopes of increasing the understanding of digesta ROP in horses [10,11,17,18,19,20]. The models described by Dhanoa et al. [14] and Pond et al. [15] have been used most frequently to describe digesta passage in horses. The Dhanoa et al. [14] model is a mechanistic model based on first order kinetics. Digesta flows through an unspecified number of compartments with decreasing compartment retention times [14]. In contrast, the stochastic model described by Pond et al. [15] increases the passage rate of an age-dependent compartment to account for an increased probability of digesta leaving a compartment based on previous residence time in the compartment. These models have not been compared with the same data, due in part to the model equations failing to converge with experimental data collected from horses. With more advanced computer applications, a thorough investigation of model fit can be conducted while also exploring the effect of dietary characteristics on passage parameters in the horse.

We hypothesized that the greater hemicellulose concentration of Coastal bermudagrass would alter digestive characteristics. The objective of this study was to compare the digestibility and MRT of Coastal bermudagrass to alfalfa and orchardgrass (*Dactylis glomerata*) hays, which are other common forages fed to horses. Mean retention time was measured using liquid and particulate phase external markers, and fecal marker excretion was modeled using previously developed equations for marker excretion by ruminants [14,15]. We hypothesized that the use of mathematical modeling would provide a greater understanding of ROP variables than TTMRT alone. Differences in total tract MRT of Coastal bermudagrass compared with alfalfa and orchardgrass hay indicate fiber chemical composition alters digesta movement in the gastrointestinal tract of the horse. Longer digesta retention of Coastal bermudagrass may be an important compensation strategy to maximize the available nutrients from slowly degraded fibers in warm-season grasses.

## 2. Materials and Methods 

All animal protocols were approved by the University of Florida Institutional Animal Care and Use Committee (201509618) under the FASS Guide for the Care and Use of Agricultural Animals in Research and Teaching [21]. This study took place from 1 July 2015 to 9 September 2015, in Gainesville, FL, USA. The mean temperature was 26.3 °C and relative humidity was 88.5% during the study period. 

Five mature Quarter Horse geldings (8 ± 3 years, 552 ± 14 kg, BCS 6.0 ± 0.4 [22], mean ± SEM) housed at the University of Florida’s Horse Teaching Unit in Gainesville, FL were used in this study. Before the start of the study, horses were fed Coastal bermudagrass hay or kept in warm-season grass pastures. Horses received routine vaccinations and anthelmintic treatment before entering the study. Farrier care was maintained during the study according to standard operating procedures of the Unit. During the study, horses were individually housed in 3.7 m × 3.7 m stalls bedded with wood shaving and provided access to 7.4 m × 18.3 m outdoor, grass-free paddocks with sand footing for 3 h each day for voluntary exercise. 

Five hays (Table 1) were used to evaluate 5 forage-based diets (Table 2). Hay was fed at 1.6% body weight (BW) (dry matter (DM) basis). Alfalfa (ALF) and orchardgrass (ORCH) hays were purchased from a commercial hay dealer (Larson Farms; Ocala, FL). Coastal bermudagrass hays were harvested in Alachua, FL at 4 weeks (CB 4), 6 weeks (CB 6), and 8 weeks (CB 8) of regrowth under similar management conditions. The CB 4 and CB 6 were second cuttings, whereas the CB 8 was a first cutting. Based on producer harvesting schedules and study timeline, 8 weeks of regrowth as a second cutting was not feasible for this study. The orchardgrass hay had a high electrolyte concentration, therefore, sodium chloride and potassium chloride were added to each diet to better balance electrolyte intake between diets. Horses were fed a vitamin/mineral pellet (0.1 to 0.125% BW, DM basis) during the evening meal to meet micronutrient requirements [23].

Diets were evaluated in a 5 × 5 Latin square design experiment. A standard 5 × 5 Latin square was randomly selected from Fisher and Yates [24]. Horses were randomly assigned to different rows and each period was considered a column. Each period lasted 14 days and consisted of a 10.5-day restricted intake phase when the ration was split into two equal-sized meals fed at 0730 and 1930 h (Table 2). On day 7, an 84-h total fecal collection that began during the evening meal was conducted to determine diet digestibility and retention time. As part of a companion study [25], horses had ad libitum access to hay for the remaining 3.5 days before the start of the next period. 

External markers were prepared and used to determine digesta MRT for each gelding. A lithium salt of Co-EDTA was prepared according to the methods of Udén et al. [27] as a marker for the liquid phase of digesta. For the particulate marker, Yb-acetate was bound to neutral detergent fiber residue according to Ringler and Lawrence [28]. Bermudagrass hay was chopped by a hammer mill until it passed through a 1.27-cm screen and then boiled in neutral detergent solution for 1 h (60 g of bermudagrass hay per liter of neutral detergent solution). Neutral detergent fiber residue was labeled at a concentration of 100 g of NDF residue/L of 0.007 M Yb solution (prepared by dissolving 2.96 g of Yb (III) acetate tetrahydrate in 1 L of distilled water) [28]. The prepared Co-EDTA was 13.7% Co (DM basis) and Yb-labeled NDF residue was 7304 mg Yb/kg DM.

On day 7 of each period, horses were fed 1.5 mg of each marker per kilogram BW with the evening meal of vitamin/mineral pellets. Marker intake was monitored and spilled feed was immediately returned to the feed bucket to ensure complete marker consumption. On average, horses consumed the markers in 14.7 min (range 9 to 30 min).

Immediately before and during fecal collections, stalls were stripped of bedding and swept clean. All voided feces were collected directly from the floor of rubber-matted stalls. In order to minimize contamination of feces with hay, dirt, and other debris and to prevent the horse from stepping in the feces, stalls were checked for fresh excreta every 15 min. Horses were removed from their stalls in 2 to 4-h intervals and temporarily placed in a stall bedded with pine shavings to allow horses to comfortably urinate. If a horse urinated in their primary stall, urine was removed with a wet-dry vacuum. Horses were hand-walked for two 15-min periods (06:00 and 20:00) each day during fecal collections.

Feces were compiled in 2-h intervals for the first 60-h following marker dosing and then in 4-h intervals from 60 to 84 h post marker dosing. Excreted feces were weighed and homogenized after each time interval with 10% of the feces retained for a 24-h composite sample and a 200-g subsample saved for marker concentration determination. Feces collected the first 12 h post marker dosing were only retained for marker concentration analysis, and feces collected from 12 h to 84 h post marker dosing were used for both marker concentration analysis and 24-h composite samples. During fecal collections, orts were collected prior to the next feeding. Orts were time-matched to 24-h fecal composites to determine diet digestibility. Fecal samples were stored at −20 °C until analysis.

Frozen fecal samples were thawed at 4 °C for 48 h. Fecal samples, representative feed samples from each total fecal collection, and orts were dried in a 60 °C forced air oven until achieving a constant weight. Samples were ground to pass a 1-mm screen using a Wiley Mill prior to laboratory analysis. 

Twenty-four-hour fecal composite samples, representative feed samples, and orts were used to determine DM, organic matter (OM), NDF, and ADF digestibility (DMD, OMD, NDFD, ADFD, respectively). Samples were dried in triplicate at 60 °C until a constant weight and then ashed at 600 °C for 8 h to calculate OM concentration. Fiber concentrations were sequentially determined using an ANKOM 200 Fiber Analyzer [29]. Heat-stable α-amylase was used in the NDF analysis of all samples. Digestibility was determined as ((Nutrient Intake − Nutrient Output)/Nutrient Intake × 100).

Marker concentrations were determined on fecal samples composited in 2- and 4-h intervals following marker dosing. Fecal samples were dried in triplicate in a 60 °C forced-air oven until a constant weight to determine DM concentration. A 0.500 g subsample was weighed and placed into a Teflon digestion vessel with 8 mL of 15.8 N nitric acid. Samples were sealed and digested for 15 min at 180 °C using a microwave-assisted acid digestion procedure (Anton-Paar, Ashland, VA, USA). Samples were allowed to cool and diluted to 25 mL. Samples were centrifuged at 1050× *g* for 15 min and the supernatant collected for determination of marker concentrations using inductively coupled plasma spectrometry (Perkin-Elmer, Inc., Shelton, CT, USA) [30,31]. The minimum element detection limit was 0.1 mg/L. Marker recovery was calculated as (Marker Excreted/Marker Dosed × 100).

Total tract MRT was calculated arithmetically according to Blaxter et al. [32] and Thielemans et al. [33]. Total tract MRT calculated according to Blaxter et al. [32] is

(1) $$\mathrm{MRT}=\frac{\sum m_{i}t_{i}}{\sum m_{i}}$$

where *m_i_* = the amount of marker in the *i*th sample (g) and *t_i_* = time from dosage of the marker to the middle of the *i*th sampling interval (h). The equation described by Thielemans et al. [33] uses the concentration of the marker in the sample and MRT is calculated as

(2) $$\mathrm{MRT}=\frac{\sum t_{i}C_{i}\Delta t_{i}}{\sum C_{i}\Delta t_{i}}$$

where *t_i_* = time from dosage of the marker to the middle of the *i*th sampling interval (h), *C_i_* = concentration of marker in the *i*th sample (mg/kg DM), and Δ*t_i_* = time interval between the middle of the *i*th and *i*th − 1 sample (h).

Fecal marker excretion data were fit with compartment models described by Dhanoa et al. [14] and Pond et al. [15]. The multicompartment model derived by Dhanoa et al. [14] is a mechanistic model based on first order kinetics where marker concentration (mg/kg DM) of the feces can be modeled as

(3) $$\mathrm{Marker}\;\mathrm{Concentration}=Ae^{-k_{1}t}e^{-\left(N-2\right)e^{-\Delta t}}$$

where *A* is a scaling parameter, *k*_1_ = rate constant for the first compartment (h^−1^), *t* = time from marker dosage (h), Δ = *k*_2_ − *k*_1_ where *k*_2_ is the rate constant for the second compartment (h^−1^, assuming *k*_2_ > *k*_1_), and *N* = the number of exponentially distributed compartments. The rate constants do not change over time; therefore, the compartments are considered age-independent (the rate digesta leaves a compartment is not influenced by past residence time). The exponentially distributed compartments described by Dhanoa et al. [14] can represent multiple sub-compartments within a larger mixing compartment. The two-compartment model featuring a γ-distribution described by Pond et al. [15] was also fit to fecal marker excretion data (mg/kg DM) as

(4) $$\mathrm{Marker}\;\mathrm{Concentration}=C_{2}\left[\delta^{n}e^{-k_{2}\left(t\right)}-e^{-\lambda_{1}t}\sum_{i=1}^{n}\frac{\delta^{i}{\left(\lambda_{1}t\right)}^{n-i}}{\left(n-i\right)!}\right]$$

where *C*_2_ = the initial concentration in the second compartment if the marker dose had been introduced into the compartment and instantaneously mixed, n = order of the γ-distribution in the first compartment, *k*_2_ = rate parameter for exponentially distributed residence times (h^−1^), *t* = time after dosing of marker (h), *λ*_1_ = rate parameter for γ-distributed residence times (h^−1^), and *δ* = *λ*_1_/(*λ*_1_ − *k*_2_). Time delay was incorporated into the Pond et al. [15] equation by substituting t for t-TT, where *t* is the time from marker dosing (h) and TT is transit time. Six orders of γ-distribution were analyzed (n = 1, 2, 3, 4, 5, 6) to test the G1G1, G2G1, G3G1, G4G1, G5G1, and G6G1 model described by Pond et al. [15]. If marker residence time is exponentially distributed (n = 1) in a compartment, the compartment is age-independent, indicating that the rate the marker leaves is not dependent on past residence time. However, if the ROP of a marker in a compartment changes over time, the compartment is considered age-dependent. Marker concentration in an age-dependent compartment can be modeled with a γ-distribution of order 2 or greater. Increasing the order of the γ-distribution alters the shape of the curve such that the emergence of marker from the compartment is slowed [15]. Curves were fit using nonlinear least squares methods in MATLAB (Version R2015a, Mathworks, Natick, MA, USA) with model parameter start values randomly assigned (Computer Code S1). Bounds for rate parameters were set between 0 and 1. 

Model parameters were used to determine total tract mean retention time (TTMRT) for each fitted equation to fecal marker excretion. For the Dhanoa et al. [14] model, TTMRT (h) was calculated as

(5) $$\mathrm{MRT}=\frac{1}{k_{1}}+\frac{1}{k_{2}}+\sum_{i=3}^{N-1}\frac{1}{k_{2}+\left(i-2\right)\left(k_{2}-k_{1}\right)},\;k_{2}>k_{1}$$

where *k*_1_ and *k*_2_ are rate parameters (h^−1^) of the first and second compartments and N is the number of exponentially distributed compartments. The term $\sum_{i=3}^{N-1}\frac{1}{k_{2}+\left(i-2\right)\left(k_{2}-k_{1}\right)}$ is said to represent the transit time (TT) of digesta markers or the time from dosing the marker to the first appearance of marker in the collected sample. Total tract MRT (h) for the Pond et al. [15] model was calculated as

(6) $$\mathrm{MRT}=\frac{n}{\lambda_{1}}+\frac{1}{k_{2}}+\mathrm{TT}$$

where *λ*_1_ is the age-dependent compartment rate constant (h^−1^), *k*_2_ is the age-independent compartment rate constant (h^−1^), n is the order of the γ-distribution, and TT is the transit time (h). The age-dependent compartment MRT (CMRT_1_) was determined by n/*λ*_1_ and the age-independent compartment MRT (CMRT_2_) was determined by 1/*k*_2_. When n = 1, *λ*_1_ is replaced by *k*_1_, and CMRT_1_ is an age-independent compartment.

Unless otherwise noted, data are presented as means ± SEM. Data were checked for normality using the Kolmogorov–Smirnov test and the Shapiro–Wilk test. Data were analyzed as a Latin square design using a mixed model ANOVA in SAS (v 3.8 SAS Studio, Cary, NC, USA). Fixed effects included dietary treatment and period, and the random effect was horse. The influence of feeding Coastal bermudagrass (CB 4, CB 6, and CB 8) compared with other hays (alfalfa and orchardgrass) on digestive variables was determined using contrasts. Statistically significant means were separated by Scheffe’s method. Model derived TTMRT was compared to arithmetic calculations using both two one-sided tests of equivalence and regression analysis. For equivalence testing, the acceptable difference was 10%. Statistical trends were defined as *p* < 0.1 and differences at *p* < 0.05.

## 3. Results

### 3.1. Diet Digestibility

One factor that affects digestibility measurements is feed refusal. Orts were collected during 26 of the 75 daily measurements of intake, most frequently when horses were fed the CB 8 diet. The mean weight of orts was 0.07 kg (DM basis). Ash concentration of hay orts ranged from 24.6% to 66.8%, indicating contamination with sand from the environment. Thus, hay ort weight was corrected by multiplying ort weight by the ratio of ort ash concentration to forage ash concentration. Orts were analyzed for nutrient composition and subtracted from nutrient intake to correct for any feed not consumed by the horses. 

Differences in fecal excretion were related to variations in diet digestibility. Horses fed Coastal bermudagrass hay diets defecated 1.4 times more frequently (*p* < 0.05) than when fed alfalfa hay (Table 3). Horses fed CB 6 and CB 8 excreted more feces (*p* < 0.05) than horses consuming ALF, ORCH, or CB 4. Dry matter and OM digestibilities were greatest (*p* < 0.05) for ALF, whereas a reduction in DMD and OMD was observed when horses were fed CB 6 and CB 8. There was a 32.0% reduction (*p* < 0.05) in NDFD and a 47.1% decrease (*p* < 0.05) in ADFD digestibility for the CB 6 and CB 8 diets compared with the other diets.

### 3.2. Fecal Marker Excretion

#### 3.2.1. Marker Excretion and Recovery

**Figure 1:**
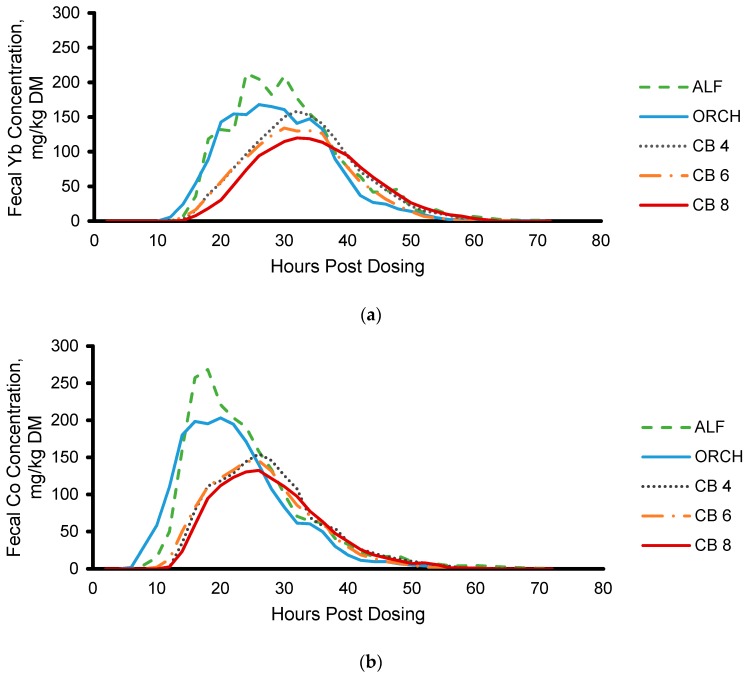
Two period moving average of fecal marker excretion of (**a**) Yb and (**b**) Co after dosing external markers (SEM = 12.4 and 13.2 mg/kg DM, respectively). Abbreviations. ALF, alfalfa; ORCH, orchardgrass; CB 4, Coastal bermudagrass 4-weeks regrowth; CB 6, Coastal bermudagrass 6-weeks regrowth; CB 8, Coastal bermudagrass 8-weeks regrowth.

Mean fecal marker excretion is presented in Figure 1. External marker concentrations were detected in feces between 5 to 13 h after feeding horses external markers. Element concentrations were below instrument detection limits by 60 h post marker dosing, thus, fecal samples were only analyzed for marker concentrations to 72 h post marker dosing. A pulsatile pattern was observed in some individual fecal marker excretion curves (Supplementary Figures S1–S5). 

Marker recovery ranged from 73.3 to 97.6% for Yb and 73.9 to 115% for Co. Particulate marker recovery did not differ by diet (Table 4). Liquid marker recovery tended to differ among diets (*p* = 0.075) with mean Co recovery greatest in the ORCH diet and lowest in the CB 4 diet. Particulate and liquid marker recovery did not differ within a horse for each period.

#### 3.2.2. Modeling Fecal Marker Excretion

Seventy six percent of model equations fit fecal excretion data for each horse within a period using initial parameter ranges and start values defined in the program code. When the model did not converge using the code, model parameter ranges were adjusted using curve fitting software in MATLAB to obtain an acceptable fit (as indicated by the R^2^ value being non-negative). 

**Figure 2:**
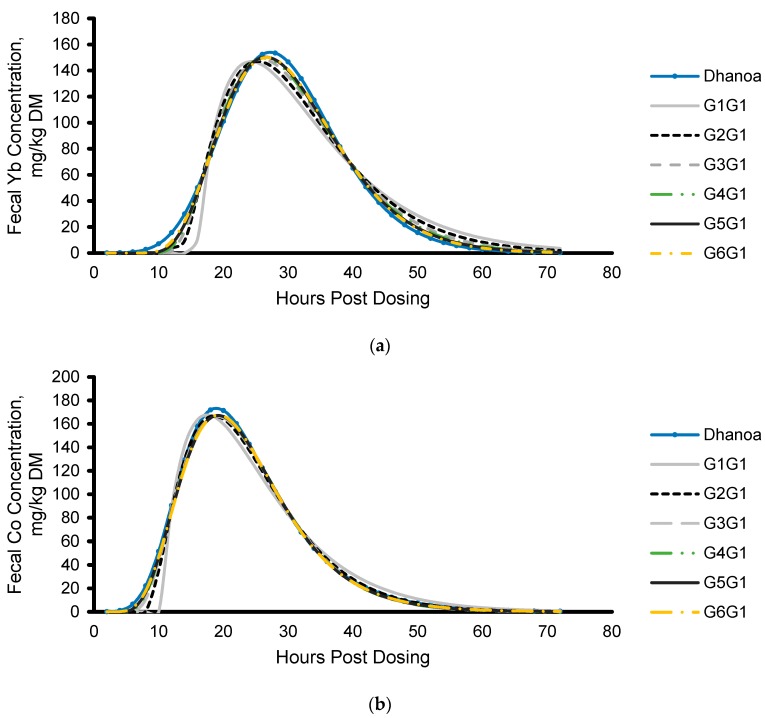
Model result derived from equations described by Dhanoa et al. [14] and Pond et al. [15] applied to all experimental data of (**a**) Yb and (**b**) Co after dosing external markers. Abbreviations. G1G1, first order two-compartment model; G2G1, second order two-compartment model; G3G1, third order two-compartment model; G4G1, fourth order two-compartment model; G5G1, fifth order two-compartment model; G6G1, sixth order two-compartment model according to Pond et al. [15].

Mean model result from all data is depicted for all equations in Figure 2. Mean model fit, parameter values, and retention time from fecal marker excretion of each observation are summarized in Table 5. Model parameters were nonzero (*p* < 0.05) for 47% of the fitted equations. The scaling parameter was less than 0 for the G1G1 model. As the order of the γ-distribution increased for the equations described by Pond et al. [15], TT, CMRT_2_, and TTMRT decreased, whereas CMRT_1_ increased. The root mean square error (RMSE) ranged from 2.932 to 42.23 and 3.369 to 29.85 for particulate and liquid fecal marker excretion, respectively (Table 5). The model described by Dhanoa et al. [14] had the lowest RMSE and Akaike’s information criterion (AIC) for the particulate and liquid phases of digesta. Among the six two-compartment γ-distributed equations described by Pond et al. [15], the G5G1 model best fit particulate marker excretion and the G4G1 equation best fit liquid marker excretion based on AIC values (Table 5). Because the AIC values increased once the order 5 and order 4 γ-gamma distributions were fit to the particulate and liquid marker excretion, fitting marker excretion to the two-compartment model was terminated at the order 6 γ-gamma distribution.

### 3.3. Digesta Mean Retention Time

#### 3.3.1. Mean Retention Time Calculated from Model Parameters

The best fitting models to describe marker excretion were used to compare digesta ROP between diets. The Dhanoa et al. [14] and Pond et al. [15] G5G1 models were used to analyze particulate phase ROP. The Dhanoa et al. [14] and Pond et al. [15] G4G1 models were used to analyze liquid digesta ROP. 

Particulate TTMRT differed by diet (*p* = 0.020 and *p* = 0.022, respectively; Table 6) when Yb marker excretion was fit to both the Dhanoa et al. [14] and Pond et al. [15] G5G1 model. Rates of passage, *k*_1_ and *k*_2_, did not differ among diets when fecal excretion was fit to equations described by Dhanoa et al. [14]; however, CMRT_1_ tended to be longer (*p* = 0.084) when horses were fed ALF compared with CB 6. Transit time calculated according to Dhanoa et al. [14] tended to be longer (*p* = 0.074) in CB 8 than ALF. Modeling particulate fecal marker excretion using the G5G1 model [15], *λ*_1_ tended to differ by diet (*p* = 0.069) with *λ*_1_ trending towards being quicker (*p* = 0.102) in ALF than CB 8. The other model parameters *k*_2_ and TT did not differ by diet. Age-dependent compartment mean retention time (CMRT_1_) was shorter (*p* = 0.014) in ALF than CB 8 and tended to be shorter (*p* = 0.065) than CB 4. There was no difference in the age-independent compartment mean retention time (CMRT_2_).

#### 3.3.2. Arithmetically Calculated Mean Retention Time

Arithmetically calculated particulate digesta MRT (Table 7) using the equation described by Blaxter et al. [32] was longer (*p* = 0.019) than when calculated according to Thielemans et al. [33], but the mean difference was only 0.2 h (30.0 ± 0.88 vs. 29.8 ± 0.88 h). Th method of calculation did not affect liquid MRT (23.4 ± 0.70 vs. 23.5 ± 0.67 h). For both equations, particulate MRT was longer (*p* < 0.001) than liquid MRT.

Particulate MRT differed by diet (*p* < 0.001) and was longer (*p* < 0.05) when horses were fed CB 8 compared with ORCH (Table 7). Liquid MRT differed by diet (*p* < 0.001). Horses fed ORCH had a shorter (*p* < 0.01) liquid MRT than when fed the Coastal bermudagrass diets. Liquid MRT differed (*p* < 0.011) between horses fed ALF compared with CB 4 and CB 8 and tended to be shorter (*p* < 0.079) than horses fed the CB 6 diet.

#### 3.3.3. Comparing Model-Derived and Arithmetically Calculated Mean Retention Time

The mean retention times calculated from Dhanoa et al. [14] and Pond et al. [15] model parameters were compared with arithmetically calculated MRT using two one-sided test of equivalence (TOST) with a 10% difference and regression analysis. Model-derived TTMRT was similar to (*p* < 0.05) MRT calculated according to the equation described by Blaxter et al. [32] or Thielemans et al. [33] for the G2G1, G3G1, G4G1, G5G1 models for the particulate phase of digesta. For the liquid phase of digesta, models described by Pond et al. [15] were similar to (*p* < 0.05) arithmetic MRT, but TTMRT calculated according to Dhanoa et al. [14] was over 2 h shorter than arithmetic MRT.

**Figure 3:**
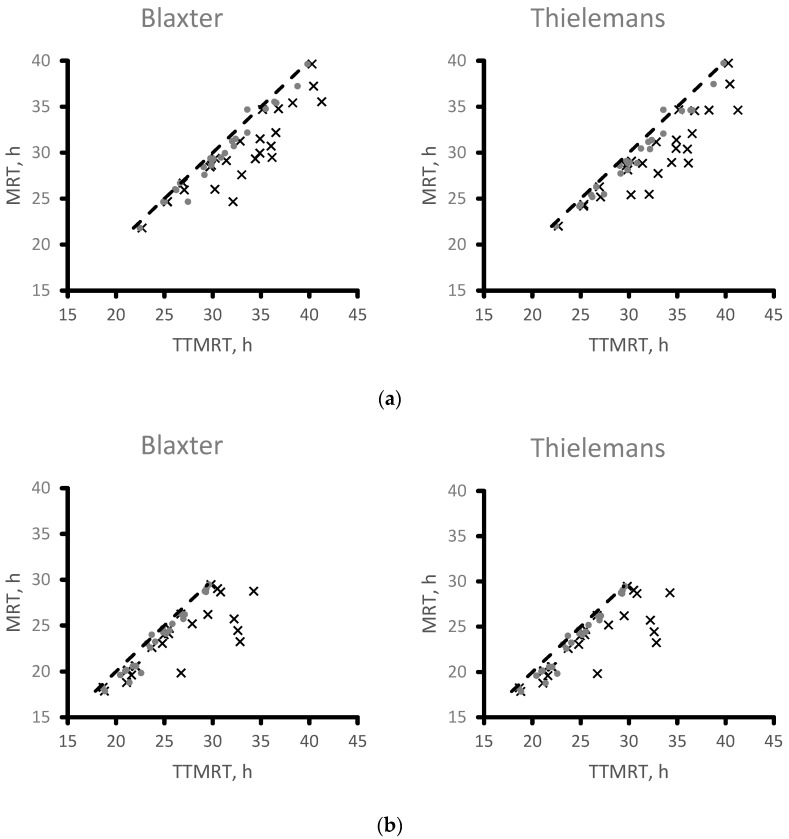
Comparison of arithmetically calculated mean retention time (MRT) calculated according to Blaxter et al. [32] and Thielemans et al. [33] compared with total tract mean retention time (TTMRT) calculated according to Dhanoa et al. [14] (×) and the best fitting two-compartment model described by Pond et al. [15] (•) for the particulate (**a**) and liquid (**b**) phase of digesta. The 1 to 1 line is denoted by the dashed line.

Arithmetically calculated MRT and model-derived TTMRT are plotted in Figure 3. For the particulate phase of digesta (Figure 2a), the equation relating TTMRT to MRT according to Blaxter et al. [32] was MRT = 0.7929x + 3.903 (RMSE = 1.980; r^2^ = 0.8071; *p* < 0.001) for TTMRT calculated according to Dhanoa et al. [14] and MRT = 0.9737x − 0.05589 (RMSE = 0.6256; r^2^ = 0.9807; *p* < 0.001) when TTMRT according to the G5G1 model described by Pond et al. [15]. When MRT was calculated according to Thielemans et al. [33], MRT = 0.7935x + 3.680 (RMSE = 1.960; r^2^ = 0.8105; *p* < 0.001) for TTMRT calculated according to Dhanoa et al. [14] and MRT = 0.9743x − 0.2772 (RMSE = 0.5592; r^2^ = 0.9846; *p* < 0.001) when TTMRT according to the G5G1 model described by Pond et al. [15]. For the liquid phase of digesta (Figure 2b), the equation relating TTMRT to MRT according to Blaxter et al. [32] was MRT = 0.6329x + 6.956 (RMSE = 2.017; r^2^ = 0.6852; *p* < 0.001) for TTMRT calculated according to Dhanoa et al. [14] and the y-intercept differed (*p* = 0.007) from zero. Mean retention time calculated according to Blaxter et al. [14] was related to TTMRT calculated according to the G4G1 model described by Pond et al. [15] as MRT = 1.064x − 2.408 (RMSE = 0.6661; r^2^ = 0.9657; *p* < 0.001) and the y-intercept differed (*p* = 0.028) from zero. When TTMRT was calculated according to Thielemans et al. [33], MRT = 0.5326x + 9.984 (RMSE = 2.565; r^2^ = 0.4879; *p* < 0.001) for TTMRT calculated according to Dhanoa et al. [14] and MRT = 0.8692x + 2.748 (RMSE = 2.129; r^2^ = 0.6474; *p* < 0.001) when TTMRT according to the G4G1 model described by Pond et al. [15]. For the regression equation relating TTMRT from the G4G1 model to MRT calculated according to Thielemans et al. [33], the y-intercept differed from zero (*p* = 0.003).

## 4. Discussion

Increasing the retention time of Coastal bermudagrass may be an important digestive strategy in horses to adapt to the more difficult-to-digest fiber particles of warm-season grasses. Greater retention time of highly fibrous, warm-season forages allows for a lengthened exposure of digesta to microbial degradation. Forage fiber composition may also influence digesta passage rate within the gastrointestinal tract, as observed when mathematically modeling fecal marker excretion when horses were fed alfalfa hay, which has an increased concentration of pectin compared to grasses.

Forage type affected DMD, with the greatest digestibility observed when horses were fed alfalfa. Several other studies have reported greater digestibility of legume hays than cool-season or warm-season grass hays [34,35,36,37,38,39,40]. The DMD of alfalfa hay in this study falls within the 54–66% range of alfalfa hay DMD reported in the literature [34,35,36,37,38,39,40]. Chemically, legume forages have greater protein and pectin concentrations and decreased insoluble fibers, allowing for a faster rate of digestion [41]. Legumes also contain a greater proportion of more easily digested mesophyll cells compared to grasses. The accumulation of lignin in alfalfa cell walls occurs primarily in alfalfa stems, whereas lignin accumulates in both grass stems and leaves during maturity [42,43]. Thus, there is a greater extent of cell wall digestion of alfalfa leaves compared to grasses [44]. 

Longer intervals of growth before harvest increased Coastal bermudagrass fiber and lignin concentrations in hay and resulted in reduced dry matter, organic matter, and fiber digestibilities. The internal girder structure of C4 forages firmly links the epidermis to vascular bundles, reducing the rate of digestion [45,46]. Akin et al. [47] reported that even typically highly digestible plant mesophyll cells were only partially degraded with increasing plant maturity in bermudagrass samples. The reported digestibility of Coastal bermudagrass hay ranges from 41–53% [34,35,48,49,50]. Although the DMD of CB 4 fell within the reported range, the CB 6 and CB 8 had reduced digestibility compared with reported values. Because fiber concentration is negatively correlated with digestibility [6], the comparatively low digestibility of CB 6 and CB 8 to other published data is surprising as the Coastal bermudagrass used in those studies often had a greater detergent fiber concentration than the hays utilized in the current study [34,48,49,50]. Although the Coastal bermudagrass harvested at four weeks regrowth had greater NDF and hemicellulose concentrations than orchardgrass and alfalfa hays, fiber digestibility was similar. However, a reduction in NDFD and ADFD was observed in the CB 6 and CB 8 diets. Phenolic compounds and hemicellulose composition changes with increasing plant cell maturity [47,51,52] likely leading to the decreased digestibility of Coastal bermudagrass harvested at longer intervals of growth. The results of the current study indicate that differences beyond plant fiber concentration, such as hemicellulose composition and lignin concentration also affect the digestibility of Coastal bermudagrass hay.

One strategy to adapt to the lower digestibility of high fiber C4 grasses such as Coastal bermudagrass is for the digesta retention time to increase. Other high fiber forages, such as oat straw, have also been reported to have increased MRT in equines [9]. Longer exposure to microbial fermentation in the equine hindgut can increase cell wall digestibility and could compensate for a slower rate of degradation of fibers in warm-season grasses [46,53,54]. This selective retention of Coastal bermudagrass hay in the gastrointestinal tract (GIT) may be due to differences in the particle size of digesta, rate of intake, or changes in GIT motility. How fiber affects gastrointestinal transit is not clearly understood in horses, people, or other mammals. One theory is that the fiber can trap water in the gastrointestinal tract, altering the way bacteria and solutes interact in the GIT [55]. In addition to luminal contents, gut motility is also driven by a range of neurohormones. Peptide YY (PYY) and glucagon-like peptide-1 (GLP-1) are important neurohormones regulating colonic motility [56], and the secretion of PYY and GLP-1 increases with the addition of fiber to diets [57,58]. Gut motility, neurotransmitters, and digesta characteristics warrant future investigation for potential mechanisms of regulating digesta transit in horses.

Differences in ROP were identified between forage types that were not apparent in TTMRT, indicating that mathematically modeling fecal marker excretion can advance the study of digesta passage in horses. Alfalfa ROP parameters derived from Dhanoa et al. [14] and Pond et al. [15] differed from other diets, indicating differences in digesta passage kinetics for a legume hay compared to grasses. For the particulate phase of digesta, *λ*_1_ was quicker and CMRT_1_ shorter in ALF than CB 8, even though no total tract differences were determined when TTMRT was calculated according to the G5G1 model. When particulate CMRT_1_ was calculated according to Dhanoa et al. [14], CMRT_1_ was longer in ALF compared with CB 6, a result also not reflected in TTMRT. When the liquid phase of digesta was modeled according to the G4G1 model, the age-dependent compartment (CMRT_1_) was shorter than the age-independent compartment when horses were fed alfalfa. In contrast, all the grass forages had longer age-dependent CMRT than age-independent CMRT. Alfalfa and other legumes have higher pectin (a soluble fiber) concentrations compared to grasses. Soluble fiber has been shown to increase retention time in the small intestine and delay gastric emptying in other species [59,60]. The effect of fiber type on digesta ROP in the horse may be better elucidated using mathematical models than total tract MRT.

In the current study, compartment models described by both Dhanoa et al. [14] and Pond et al. [15] adequately fit equine marker excretion using non-linear least squares methods by modifying model parameter start values and bounds of rate parameters. Both one- and two-compartment models have been used in the recent literature, with the best fitting model depending on the study. Equations described by both Dhanoa et al. [14] and Pond et al. [15] have failed to produce a solution of acceptable model fit due to lack of convergence between experimental data and the model [10,19,61]. However, computing power and collaboration with computer scientists and modelers can greatly reduce the likelihood of models failing to converge with experimental data. Future studies incorporating mathematical modeling into digesta ROP studies will help to identify the best ways to describe digesta passage in the equine GIT. 

Although using the equation described by Dhanoa et al. [14] resulted in an improved fit compared with the Pond et al. [15] models based on AIC values, TTMRT from the best fitting Pond et al. [15] models were more similar to arithmetically calculated MRT. The mathematical basis of these two models differ. The equation described by Dhanoa et al. [14] represents an unspecified number of exponentially distributed compartments, whereas the Pond et al. [15] equations used in the current study represented two distinct compartments plus transit time. The first two compartments in the Dhanoa et al. [14] model (CMRT_1_ and CMRT_2_) have the longest retention time, and the remaining compartments are summed to determine transit time. Transit time is often defined as the amount of time from marker dosing to the first appearance of marker in the feces, but Ellis et al. [62] described transit time in the Dhanoa et al. [14] model as the sum of time in the remaining system of mixing compartments. The difference in transit time definition may be part of the discrepancy between transit time calculated from the different model equations. Additionally, the Dhanoa et al. [14] model may represent a set of exponentially distributed compartments that are within a larger mixing structure. The lack of agreement between arithmetic MRT and TTMRT calculated by the Dhanoa et al. [14] model may be explained by these differences.

The use of mathematical models could be further enhanced if theoretical compartments could be correlated to anatomical sections of the GIT. Moore-Colyer et al. [10] hypothesized that the two compartments of the Pond et al. [15] model represented the large colon for the age-dependent compartment and the cecum for the age-independent compartment because retention time in the colon is longer than the retention time in the cecum [63]. Transit time was hypothesized to represent residence time in the remaining structures of the GIT (i.e., stomach and small intestine). In a previous study using similar methodology, modeling fecal marker excretion when horses were fed mainly forage diets resulted in a CMRT similar to MRT in the cecum and colon [64,65]. However, Murray et al. [19] rejected the hypothesis that the age-dependent compartment was the colon because they observed a longer retention time in the age-independent compartment. The longer retention time of the age-independent compartment by Murray et al. [19] may be due to the diet (alfalfa and sugar beet pulp, which are high in soluble fiber), as observed in this study. Thus, diet may have a greater influence on compartment mean retention time than connections with anatomical compartments. Overall, mathematical models show promise to describe passage kinetics in horses, but the physiological relevance of compartment retention times remains unclear.

## 5. Conclusions

In conclusion, the horse appears to adopt a digestive strategy to decrease the rate of passage of digesta when fed warm-season grass forages. Increasing the retention time allows for fiber particles with greater hemicellulose and lignin concentrations to be exposed to microbial fermentation longer. Because warm-season forages have slower rates of degradation, this change in retention time allows the horse to maximize potential nutrients obtained from the diet. Using mathematical models further characterized differences in digesta ROP between forages which were not apparent when evaluating total tract MRT alone. Minor discrepancies between models and arithmetically calculated MRT were observed and should be resolved for future use. Nonetheless, mathematical modeling should be incorporated into future equine nutrition research to expand knowledge on digesta passage and equine science in general. 

## Figures and Tables

**Table 1:** Nutrient composition of feedstuffs.

Nutrient ^a^	Alfalfa	Orchardgrass	Coastal 4 Weeks	Coastal 6 Weeks	Coastal 8 Weeks	Vit/Min Suppl 1 ^b^	Vit/Min Suppl 2 ^c^
DM, %	88.4	90.9	90.0	91.8	91.6	89.4	90.5
DE ^d^, Mcal/kg	2.50	2.09	1.95	1.90	1.85	2.76	3.31
CP, %	23.2	11.5	18.5	12.7	12.6	15.3	37.2
NDF, %	37.7	57.2	67.5	70.9	73.3	43.3	16.9
ADF, %	29.5	42.0	32.7	34.7	35.1	25.9	8.4
ADL, %	8.3	2.8	4.6	5.0	6.0	n.m.	n.m.
Starch, %	1.3	0.2	1.6	1.6	2.5	n.m.	n.m.
ESC, %	5.9	9.6	4.3	4.4	4.4	n.m.	n.m.
WSC, %	6.3	12.5	3.6	4.3	4.8	n.m.	n.m.
Ca, %	1.58	0.32	0.57	0.39	0.38	1.31	3.12
P, %	0.24	0.23	0.30	0.27	0.18	1.78	1.19
Na, %	0.067	0.44	0.067	0.023	0.12	0.23	0.40
K, %	2.33	2.12	1.58	1.74	0.82	1.03	1.60
Cl, %	0.93	1.56	0.45	0.33	0.17	0.60	0.75
uNDFom, % ^e^	20.9	11.6	21.4	25.8	38.6	n.m.	n.m.

^a^ Nutrient composition of forages analyzed by NIRS at Dairy One Inc. (Ithaca, NY, USA). ^b^ Gro-n-Win alfa (Buckeye Nutrition, Dalton, OH, USA) analyzed by wet chemistry at Dairy One Inc. (Ithaca, NY, USA). ^c^ Equalizer (Seminole Feed, Ocala, FL, USA) analyzed by wet chemistry at Dairy One Inc. (Ithaca, NY, USA). ^d^ Digestible energy calculated according to Pagan [26]. ^e^ Undigestible NDF (ash-free) determined after 240-h in vitro incubation by Dairyland Laboratories (Arcadia, WI, USA). All values are on a 100% DM basis except DM. n.m. not measured.

**Table 2:** Diet composition and nutrient intake of experimental diets ^1^.

Item	ALF	ORCH	CB 4	CB 6	CB 8
**Ingredient, % DMI**					
**Alfalfa**	93.7				
**Orchardgrass**		92.8			
**Coastal Bermuda, 4 weeks**			91.8		
**Coastal Bermuda, 6 weeks**				91.8	
**Coastal Bermuda, 8 weeks**					91.4
**Vit/Min Suppl 1 ^a^**	5.9				
**Vit/Min Suppl 2 ^b^**		7.2	7.2	7.2	7.1
**Sodium Chloride**	0.4		0.3	0.4	0.2
**Potassium Chloride**			0.7	0.6	1.3
**Daily Intake**					
**DM, % BW**	1.71	1.73	1.74	1.74	1.75
**DE ^c^, Mcal/kg BW**	0.043	0.038	0.035	0.35	0.034
**CP, g/kg BW**	3.87	2.31	3.43	2.50	2.48
**NDF, g/kg BW**	6.47	9.36	11.01	11.56	11.94
**ADF, g/kg BW**	4.98	6.83	5.34	5.65	5.72
**Ca, mg/kg BW**	265.9	90.23	130.2	101.4	99.82
**P, mg/kg BW**	56.2	51.69	62.89	58.09	43.69
**K, mg/kg BW**	383.1	359.2	332.8	348.4	261.2
**Na, mg/kg BW**	39.3	75.03	37.92	30.88	35.6
**Cl, mg/kg BW**	197.8	359.2	174.5	145.9	159.4

^1^ Abbreviations. ALF, alfalfa; ORCH, orchardgrass; CB 4, Coastal bermudagrass 4-weeks regrowth; CB 6, Coastal bermudagrass 6-weeks regrowth; CB 8, Coastal bermudagrass 8-weeks regrowth. ^a^ Gro-n-Win alfa (Buckeye Nutrition, Dalton, OH, USA). ^b^ Equalizer (Seminole Feeds, Ocala, FL, USA). ^c^ Digestible energy calculated according to Pagan [26].

**Table 3:** Fecal excretion and diet digestibility of five experimental diets ^1^ (*n* = 5).

Variable	ALF	ORCH	CB 4	CB 6	CB 8	SEM	Diet ^2^ *p*-Value	Contrast ^3^ *p*-Value
Defecation Frequency, times/d	10.0 ^c^	11.5 ^b,c^	14.1 ^a,b^	15.3 ^a^	14.0 a,b	0.5	<0.001	<0.001
Fecal Excretion, kg DM/d	3.55 ^d^	4.41 ^c^	5.04 ^b^	5.84 ^a^	5.87 ^a^	0.20	<0.001	<0.001
Fecal DM, %	19.7	20.9	20.0	20.5	22.5	0.42	0.074	0.255
Urination Frequency, times/d	10.6	10.6	8.7	8.3	10.7	0.58	0.324	0.161
**Digestibility, %**								
DM	62.1 ^a^	51.2 ^b^	47.2 ^b^	36.0 ^c^	36.8 ^c^	2.1	<0.001	<0.001
OM	63.1 ^a^	52.3 ^b^	46.8 ^c^	37.3 ^d^	37.6 ^d^	2.1	<0.001	<0.001
NDF	43.1 ^a^	42.4 ^a^	46.2 ^a^	31.1 ^b^	31.8 ^b^	1.7	<0.001	<0.001
ADF	40.2 ^a^	39.8 ^a^	39.8 ^a^	23.9 ^b^	24.3 ^b^	1.9	<0.001	<0.001

^1^ Abbreviations. ALF, alfalfa; ORCH, orchardgrass; CB 4, Coastal bermudagrass 4-weeks regrowth; CB 6, Coastal bermudagrass 6-weeks regrowth; CB 8, Coastal bermudagrass 8-weeks regrowth; SEM, standard error of the mean. ^2^ Main effect of diet. ^3^ Contrast between Coastal bermudagrass (CB 4, CB 6, CB 8) and other diets (ALF, ORCH). ^a,b,c,d^ Means with unlike superscripts differ (*p* < 0.05).

**Table 4:** Particulate (Yb) and liquid (Co) marker recovery ^1^ (*n* = 5).

Variable	ALF	ORCH	CB 4	CB 6	CB 8	SEM	Diet ^2^ *p*-Value	Contrast ^3^ *p*-Value
Particulate, %	80.6	86.3	85.5	82.5	85.0	1.44	0.549	0.948
Liquid, %	85.5	95.1	79.4	80.3	82.6	2.17	0.075	0.025

^1^ Abbreviations. ALF, alfalfa; ORCH, orchardgrass; CB 4, Coastal bermudagrass 4-weeks regrowth; CB 6, Coastal bermudagrass 6-weeks regrowth; CB 8, Coastal bermudagrass 8-weeks regrowth; SEM, standard error of the mean. ^2^ Main effect of diet. ^3^ Contrast between Coastal bermudagrass (CB 4, CB 6, CB 8) and other diets (ALF, ORCH).

**Table 5:** Model goodness of fit, parameters, and retention time of equations used to fit particulate (Yb) and liquid (Co) fecal marker excretion (diets combined, *N* = 25; means ± SE).

Variable	Dhanoa ^a^	G1G1 ^b^	G2G1 ^b^	G3G1 ^b^	G4G1 ^b^	G5G1 ^b^	G6G1 ^b^
Particulate							
RMSE	11.70	20.48	16.30	14.83	14.02	12.43	12.91
AIC	128.8	163.1	148.1	142.3	138.7	134.3	137.0
Models with Nonzero Rate Parameters, %	4	0	20	72	76	84	92
*A*	2.68 × 10^11^ ± 2.65 × 10^11^						
*C*, mg Yb/kg digesta		−476 ± 26.6	636 ± 39.6	761 ± 49.6	856 ± 57.6	901 ± 56.3	916 ± 63.0
*k*_1_, h^−1^	0.265 ± 0.017	0.133 ± 0.007					
*k*_2_, h^−1^	0.355 ± 0.011	0.142 ± 0.008	0.187 ± 0.006	0.223 ± 0.008	0.253 ± 0.008	0.271 ± 0.009	0.277± 0.012
*λ*_1_, h^−1^			0.192 ± 0.007	0.235 ± 0.011	0.279 ± 0.014	0.321 ± 0.018	0.370 ± 0.024
N	48 ± 12.9						
TT, h	25.8 ± 1.15	17.5 ± 0.71	15.6 ± 0.64	13.4 ± 0.69	11.9 ± 0.66	10.6 ± 0.62	9.50 ± 0.61
CMRT_1_, h	4.20 ± 0.29	8.01 ± 0.40	10.7 ± 0.34	13.3 ± 0.47	15.0 ± 0.56	16.4 ± 0.63	17.4 ± 0.77
CMRT_2_, h	2.89 ± 0.096	7.48 ± 0.33	5.46 ± 0.15	4.61 ± 0.17	4.07 ± 0.17	3.82 ± 0.18	3.87 ± 0.26
TTMRT, h	32.9 ± 1.00	33.0 ± 0.99	31.7 ± 0.93	31.3 ± 0.92	31.0 ± 0.90	30.8 ± 0.90	30.8 ± 0.88
Liquid							
RMSE	13.88	17.51	15.57	14.95	14.04	12.43	14.01
AIC	141.1	154.6	147.2	145.0	142.6	143.0	143.5
Models with Nonzero Rate Parameters, %	16	0	52	76	80	84	84
*A*	8.00 × 10^8^ ± 4.33 × 10^8^						
*C*, mg Co/kg digesta		−470 ± 26.1	527 ± 21.4	582 ± 33.7	630 ± 42.6	642 ± 47.6	639 ± 66.3
*k*_1_, h^−1^	0.201 ± 0.020	0.202 ± 0.031					
*k*_2_, h^−1^	0.443 ± 0.045	0.144 ± 0.006	0.166 ± 0.008	0.184 ± 0.011	0.202 ± 0.015	0.206 ± 0.017	0.213 ± 0.020
*λ*_1_, h^−1^			0.333 ± 0.055	0.430 ± 0.072	0.517 ± 0.086	0.593 ± 0.097	0.655 ± 0.105
N	8.34 × 10^4^ ± 83191						
TT, h	17.3 ± 1.27	11.8 ± 0.48	10.1 ± 0.41	8.49 ± 0.38	7.47 ± 0.39	6.32 ± 0.43	5.41 ± 0.46
CMRT_1_, h	6.18 ± 0.53	6.20 ± 0.40	8.12 ± 0.64	9.81 ± 0.85	10.9 ± 0.97	12.0 ± 1.10	12.8 ± 1.17
CMRT_2_, h	2.57 ± 0.15	7.23 ± 0.29	6.39 ± 0.34	6.09 ± 0.44	5.85 ± 0.51	5.86 ± 0.52	5.87 ± 0.55
TTMRT, h	26.0 ± 0.92	25.2 ± 0.79	24.6 ± 0.71	24.4 ± 0.68	24.3 ± 0.65	24.1 ± 0.63	24.1 ± 0.63

^a^ Dhanoa et al. [14] ^b^ Pond et al. [15]. Abbreviations. G1G1, first order two-compartment model; G2G1, second order two-compartment model; G3G1, third order two-compartment model; G4G1, fourth order two-compartment model; G5G1, fifth order two-compartment model; G6G1, sixth order two-compartment model according to Pond et al. [15]; RMSE, root mean square error; AIC, Akaike’s information criterion; TT, transit time; CMRT, compartment mean retention time; TTMRT, total tract mean retention time.

**Table 6:** Model parameters and compartment retention times for particulate (Yb) and liquid (Co) marker fecal excretion ^1^ (*n* = 5).

Item	ALF	ORCH	CB 4	CB 6	CB 8	SEM	Diet ^2^ *p*-Value	Contrast ^3^ *p*-Value
Particulate								
Dhanoa A								
*A*	2.4 × 10^9^	3.0 × 10^9^	1.3 × 10^12^	3.1 × 10^9^	3.5 × 10^9^	4.3 × 10^8^		
*k*_1_, h^−1^	0.210	0.287	0.248	0.294	0.259	0.0536	0.223	0.239
*k*_2_, h^−1^	0.357	0.380	0.336	0.372	0.328	0.0450	0.338	0.312
N	107	34	29	36	33	83191	0.179	0.054
TT, h	22.0 ^y^	23.5 ^x,y^	27.9 ^x,y^	26.9 ^x,y^	28.7 ^x^	1.27	0.035	0.004
CMRT_1_, h	5.59 ^x^	3.77 ^x,y^	3.82 ^x,y^	3.63 ^y^	4.17 ^x,y^	0.532	0.049	0.051
CMRT_2_, h	2.92	2.68	3.03	2.70	3.12	0.148	0.303	0.479
TTMRT, h	30.5 ^y^	30.0 ^y^	34.7 ^x,y^	33.2 ^x,y^	35.9 ^x^	0.921	0.020	0.003
G5G1 ^B^								
*C*, mg Yb/kg digesta	1134	1007	906	780	681	56.3		
*λ*_1_, h^−1^	0.396	0.342	0.277	0.319	0.272	0.018	0.069	0.014
*k*_2_, h^−1^	0.249	0.279	0.280	0.274	0.272	0.009	0.751	0.489
TT, h	11.4	9.02	10.3	11.1	11.4	0.616	0.362	0.948
CMRT_1_, h	13.6 ^b,y^	15.4 ^a,b^	18.2 ^a,b,x^	16.1 ^a,b^	18.5 ^a^	0.634	0.008	0.002
CMRT_2_, h	4.47	3.64	3.59	3.71	3.71	0.182	0.508	0.218
TTMRT, h	29.5 ^a,b^	28.1 ^b^	32.1 ^a,b^	30.9 ^a,b^	33.6 ^a^	0.900	0.022	0.007
Liquid								
Dhanoa ^A^								
*A*	1919	3.0 × 10^9^	1.5 × 10^9^	1.3 × 10^9^	9.8 × 10^7^	2.7 × 10^11^		
*k*_1_, h^−1^	0.106 ^x^	0.234	0.145	0.111 ^y^	0.120	0.0165	0.042	0.023
*k*_2_, h^−1^	0.708 ^x^	0.440	0.373	0.376	0.320 ^y^	0.0108	0.042	0.016
N	4.2 × 10^5^	50	49	33	31.2	13	0.661	0.075
TT, h	11.3 ^b^	13.6 ^a,b^	19.9 ^a^	20.7 ^a^	20.7 ^a^	1.14	0.005	<0.001
CMRT_1_, h	9.52 ^a^	6.14 ^b^	5.57 ^b^	4.0 ^b^	5.58 ^b^	0.291	<0.001	<0.001
CMRT_2_, h	1.75 ^b^	2.48 ^a,b^	2.75 ^a,b^	2.70 ^a,b^	3.17 ^a^	0.096	0.009	0.005
TTMRT, h	22.6 ^c,z^	22.3 ^b,c,y,z^	28.2 ^a,b,x,y^	27.5 ^a,b,c,x^	29.5 ^a^	1.00	0.002	<0.001
G4G1 ^B^								
*C*, mg Yb/kg digesta	465	801	614	704	565	43		
*λ*_1_, h^−1^	1.095 ^a,x^	0.491 ^a,b,y^	0.359 ^b^	0.329 ^b^	0.310 ^b^	0.0856	0.002	0.001
*k*_2_, h^−1^	0.107 ^b,y^	0.216 ^a,b,x^	0.203 ^a,b^	0.247 ^a^	0.236 ^a,b,x^	0.0152	0.010	0.012
TT, h	8.42 ^x^	5.45 ^y^	8.15	7.34	8.00	0.391	0.042	0.194
CMRT_1_, h	4.71 ^b^	10.3 ^a^	12.6 ^a^	13.4 ^a^	13.6 ^a^	0.973	<0.001	<0.001
CMRT_2_, h	9.44 ^a^	5.32 ^b^	5.61 ^b^	4.20 ^b^	4.69 ^b^	0.513	0.001	0.002
TTMRT, h	22.6 ^b,c^	21.0 ^c^	26.4 ^a^	25.0 ^a,b^	26.3 ^a^	0.650	<0.001	<0.001

^1^ Abbreviations. ALF, alfalfa; ORCH, orchardgrass; CB 4, Coastal bermudagrass 4-weeks regrowth; CB 6, Coastal bermudagrass 6-weeks regrowth; CB 8, Coastal bermudagrass 8-weeks regrowth; SEM, standard error of the mean; RMSE, root mean square error; AIC, Akaike’s information criterion; TT, transit time; CMRT, compartment mean retention time; TTMRT, total tract mean retention time. ^2^ Main effect of diet. ^3^ Contrast between Coastall bermudagrass (CB 4, CB 6, CB 8) and other diets (ALF, ORCH). ^A^ Dhanoa et al. [14]. ^B^ Pond et al. [15]. ^a,b,c^ Means with unlike superscripts differ (*p* < 0.05). ^x,y,z^ Means with unlike superscripts tend to differ (*p* < 0.1).

**Table 7:** Mean retention time (h) of particulate digesta measured with Yb-NDF and Co-EDTA external markers ^1^ (*n* = 5).

Equation	ALF	ORCH	CB 4	CB 6	CB 8	SEM	Diet ^2^ *p*-Value	Contrast ^3^ *p*-Value
Blaxter et al. [32]								
Particulate	29.2 ^a,b^	26.9 ^b^	31.1 ^a,b^	30.0 ^a,b^	32.7 ^a^	0.88	0.010	0.006
Liquid	21.3 ^b,c,y^	20.1 ^c^	25.7 ^a^	24.3 ^a,b,x^	25.9 ^a^	0.70	<0.001	<0.001
Thielemans et al. [33]								
Particulate	28.9 ^a,b^	27.0 ^b^	30.4 ^a,b^	29.6 ^a,b^	32.6 ^a^	0.88	0.010	0.008
Liquid	21.2 ^b,c,y^	20.6 ^c^	25.4 ^a^	24.1 ^a,b,x^	25.9 ^a^	0.67	<0.001	<0.001

^1^ Abbreviations. ALF, alfalfa; ORCH, orchardgrass; CB 4, Coastal bermudagrass 4-weeks regrowth; CB 6, Coastal bermudagrass 6-weeks regrowth; CB 8, Coastal bermudagrass 8-weeks regrowth; SEM, standard error of the mean. ^2^ Main effect of diet. ^3^ Contrast between Coastal bermudagrass (CB 4, CB 6, CB 8) and other diets (ALF, ORCH). ^a,b,c^ Means with unlike superscripts differ (*p* < 0.05). ^x,y^ Means with unlike superscripts tend to differ (*p* < 0.10).

## Supplementary Materials

The following are available online at https://www.mdpi.com/2076-2615/9/12/1148/s1, Computer Code S1: Matlab Model Fit; Supplementary Figures S1–S5: Individual Horse Marker Excretion Within Diet.
