# Supplementary material for: Digestibility and Retention Time of Coastal Bermudagrass (Cynodon dactylon) Hay by Horses
Source: Animals (Basel). 2019 Dec 14;9(12):1148. doi: 10.3390/ani9121148 (PMC6940996; doi:10.3390/ani9121148)
Supplement: Supplementary file 1 [file animals-09-01148-s001.zip › Supplementary - Computer Code S1.docx]

Computer Code S1

Matlab Model Fit

function [fitresult, gof] = fecal_curve_fit(time_post_dose, yb_concentration, co_concentration)

%fecal_curve_fit(HORSE_P#_H_POST,HORSE_P#_YB,HORSE_P#_CO)

% Create fits.

%

% Data for 'Exponential Particulate' fit:

% X Input : time_post_dose

% Y Output: marker_concentration

% Data for 'Gamma 3 Particulate' fit:

% X Input : time_post_dose

% Y Output: marker_concentration

% Data for 'Gamma 2 Particulate' fit:

% X Input : time_post_dose

% Y Output: marker_concentration

% Data for 'Gamma 4 Particulate' fit:

% X Input : time_post_dose

% Y Output: marker_concentration

% Data for 'Gamma 5 Particulate' fit:

% X Input : time_post_dose

% Y Output: marker_concentration

% Data for 'Gamma 2 Liquid' fit:

% X Input : time_post_dose

% Y Output: marker_concentration

% Data for 'Exponential Liquid' fit:

% X Input : time_post_dose

% Y Output: marker_concentration

% Data for 'Gamma 3 Liquid' fit:

% X Input : time_post_dose

% Y Output: marker_concentration

% Data for 'Gamma 4 Liquid' fit:

% X Input : time_post_dose

% Y Output: marker_concentration

% Data for 'Gamma 5 Liquid' fit:

% X Input : time_post_dose

% Y Output: marker_concentration

% Data for 'Gamma 6 Liquid' fit:

% X Input : time_post_dose

% Y Output: marker_concentration

% Data for 'Dhanoa Particulate' fit:

% X Input : time_post_dose

% Y Output: marker_concentration

% Data for 'Dhanoa Liquid' fit:

% X Input : time_post_dose

% Y Output: marker_concentration

% Data for 'Gamma 6 Particulate' fit:

% X Input : time_post_dose

% Y Output: marker_concentration

% Data for 'Gamma 6 Liquid' fit:

% X Input : time_post_dose

% Y Output: marker_concentration

% Output:

% fitresult : a cell-array of fit objects representing the fits.

% gof : structure array with goodness-of fit info.

%

% See also FIT, CFIT, SFIT.

%% Initialization.

% Initialize arrays to store fits and goodness-of-fit.

fitresult = cell( 14, 1 );

%fitresult = struct('model',cell(14,1), 'a',[],'c',[],'d',[],'X0',[])

gof = struct( 'sse', cell( 14, 1 ), ...

'rsquare', [], 'dfe', [], 'adjrsquare', [], 'rmse', [] );

%Edit data so values below PQL (0.1 mg/L -> 5 mg/kg DM) are removed.

yb_concentration(yb_concentration<5)=0;

co_concentration(co_concentration<5)=0;

%% Fit: 'Exponential Particulate'.

[xData, yData] = prepareCurveData( time_post_dose, yb_concentration );

% Set up fittype and options.

ft = fittype( '0*(x<x0)+[a*d*[(exp(-c*(x-x0))-exp(-d*(x-x0)))/(c-d)]].*(x>=x0)', 'independent', 'x', 'dependent', 'y' );

opts = fitoptions( 'Method', 'NonlinearLeastSquares' );

opts.Display = 'Off';

opts.Lower = [-Inf 0 0 0];

opts.MaxFunEvals = 3000;

opts.StartPoint = [0.546805718738968 0.521135830804001 0.231594386708524 0.488897743920167];

opts.Upper = [Inf 1 1 Inf];

% Fit model to data.

[fitresult{1}, gof(1)] = fit( xData, yData, ft, opts );

% Plot fit with data.

figure( 'Name', 'Exponential Particulate' );

h = plot( fitresult{1}, xData, yData );

legend( h, 'yb_conc vs. time_post', 'Gamma 6 Particulate', 'Location', 'NorthEast' );

% Label axes

xlabel time_post

ylabel yb_conc

grid on

%% Fit: 'Gamma 3 Particulate'.

[xData, yData] = prepareCurveData( time_post_dose, yb_concentration );

% Set up fittype and options.

ft = fittype( '0*(x<x0)+[a*[(d/(d-c))^3*exp(-c*(x-x0))-exp(-d*(x-x0))*((d/(d-c))^3+(d/(d-c))^2*d*(x-x0)+((d/(d-c))*d^2*(x-x0)^2)/2)]].*(x>=x0)', 'independent', 'x', 'dependent', 'y' );

opts = fitoptions( 'Method', 'NonlinearLeastSquares' );

opts.Display = 'Off';

opts.Lower = [-Inf 0 0 -Inf];

opts.MaxFunEvals = 1000000;

opts.StartPoint = [0.65375734866856 0.49417393663927 0.779051723231275 0.715037078400694];

opts.Upper = [Inf 1 1 Inf];

% Fit model to data.

[fitresult{2}, gof(2)] = fit( xData, yData, ft, opts );

% Plot fit with data.

figure( 'Name', 'Gamma 3 Particulate' );

h = plot( fitresult{2}, xData, yData );

legend( h, 'yb_conc vs. time_post', 'Gamma 6 Particulate', 'Location', 'NorthEast' );

% Label axes

xlabel time_post

ylabel yb_conc

grid on

%% Fit: 'Gamma 2 Particulate P1'.

[xData, yData] = prepareCurveData( time_post_dose, yb_concentration );

% Set up fittype and options.

ft = fittype( '0*(x<x0)+[a*[(d/(d-c))^2*exp(-c*(x-x0))-exp(-d*(x-x0))*((d/(d-c))^2+(d/(d-c))*d*(x-x0))]]*(x>=x0)', 'independent', 'x', 'dependent', 'y' );

opts = fitoptions( 'Method', 'NonlinearLeastSquares' );

opts.Display = 'Off';

opts.Lower = [-Inf 0 0 -Inf];

opts.StartPoint = [0.0292202775621463 0.928854139478045 0.730330862855453 0.488608973803579];

opts.Upper = [Inf 1 1 30];

% Fit model to data.

[fitresult{3}, gof(3)] = fit( xData, yData, ft, opts );

% Plot fit with data.

figure( 'Name', 'Gamma 2 Particulate P1' );

h = plot( fitresult{3}, xData, yData );

legend( h, 'yb_conc vs. time_post', 'Gamma 6 Particulate', 'Location', 'NorthEast' );

% Label axes

xlabel time_post

ylabel yb_conc

grid on

%% Fit: 'Gamma 4 Particulate P1'.

[xData, yData] = prepareCurveData( time_post_dose, yb_concentration );

% Set up fittype and options.

ft = fittype( '0*(x<x0)+[a*[(d/(d-c))^4*exp(-c*(x-x0))-exp(-d*(x-x0))*((d/(d-c))^4+(d/(d-c))^3*d*(x-x0)+((d/(d-c))^2*d^2*(x-x0)^2)/2+((d/(d-c))*d^3*(x-x0)^3)/6)]].*(x>=x0)', 'independent', 'x', 'dependent', 'y' );

opts = fitoptions( 'Method', 'NonlinearLeastSquares' );

opts.Display = 'Off';

opts.MaxFunEvals = 10000;

opts.Lower = [-Inf 0 0 -Inf];

opts.StartPoint = [0.435698684103899 0.311102286650413 0.923379642103244 0.430207391329584];

opts.Upper = [Inf 1 1 Inf];

% Fit model to data.

[fitresult{4}, gof(4)] = fit( xData, yData, ft, opts );

% Plot fit with data.

figure( 'Name', 'Gamma 4 Particulate P1' );

h = plot( fitresult{4}, xData, yData );

legend( h, 'yb_conc vs. time_post', 'Gamma 6 Particulate', 'Location', 'NorthEast' );

% Label axes

xlabel time_post

ylabel yb_conc

grid on

%% Fit: 'Gamma 5 Particulate P1'.

[xData, yData] = prepareCurveData( time_post_dose, yb_concentration );

% Set up fittype and options.

ft = fittype( '0*(x<x0)+[a*[(d/(d-c))^5*exp(-c*(x-x0))-exp(-d*(x-x0))*((d/(d-c))^5+(d/(d-c))^4*d*(x-x0)+((d/(d-c))^3*d^2*(x-x0)^2)/2+((d/(d-c))^2*d^3*(x-x0)^3)/6+((d/(d-c))*d^4*(x-x0)^4)/24)]].*(x>=x0)', 'independent', 'x', 'dependent', 'y' );

opts = fitoptions( 'Method', 'NonlinearLeastSquares' );

opts.Display = 'Off';

opts.Lower = [-Inf 0 0 -Inf];

opts.MaxFunEvals = 1000;

opts.StartPoint = [0.301246330279491 0.470923348517591 0.230488160211558 0.844308792695389];

opts.Upper = [Inf 1 1 Inf];

% Fit model to data.

[fitresult{5}, gof(5)] = fit( xData, yData, ft, opts );

% Plot fit with data.

figure( 'Name', 'Gamma 5 Particulate P1' );

h = plot( fitresult{5}, xData, yData );

legend( h, 'yb_conc vs. time_post', 'Gamma 6 Particulate', 'Location', 'NorthEast' );

% Label axes

xlabel time_post

ylabel yb_conc

grid on

%% Fit: 'Gamma 2 Liquid P1'.

[xData, yData] = prepareCurveData( time_post_dose, co_concentration );

% Set up fittype and options.

ft = fittype( '0*(x<x0)+[a*[(d/(d-c))^2*exp(-c*(x-x0))-exp(-d*(x-x0))*((d/(d-c))^2+(d/(d-c))*d*(x-x0))]]*(x>=x0)', 'independent', 'x', 'dependent', 'y' );

opts = fitoptions( 'Method', 'NonlinearLeastSquares' );

opts.Display = 'Off';

opts.Lower = [-Inf 0 0 -Inf];

opts.StartPoint = [0.796183873585212 0.0987122786555743 0.261871183870716 0.335356839962797];

opts.Upper = [Inf 1 1 Inf];

% Fit model to data.

[fitresult{6}, gof(6)] = fit( xData, yData, ft, opts );

% Plot fit with data.

figure( 'Name', 'Gamma 2 Liquid P1' );

h = plot( fitresult{6}, xData, yData );

legend( h, 'yb_conc vs. time_post', 'Gamma 6 Particulate', 'Location', 'NorthEast' );

% Label axes

xlabel time_post

ylabel yb_conc

grid on

%% Fit: 'Exponential Liquid P1'.

[xData, yData] = prepareCurveData( time_post_dose, co_concentration );

% Set up fittype and options.

ft = fittype( '0*(x<x0)+[a*d*[(exp(-c*(x-x0))-exp(-d*(x-x0)))/(c-d)]].*(x>=x0)', 'independent', 'x', 'dependent', 'y' );

opts = fitoptions( 'Method', 'NonlinearLeastSquares' );

opts.Display = 'Off';

opts.Lower = [-Inf 0 0 -Inf];

opts.MaxFunEvals = 100000;

opts.MaxIter = 40000;

opts.StartPoint = [0.987982003161633 0.0377388662395521 0.885168008202475 0.913286827639239];

opts.Upper = [Inf 1 1 Inf];

% Fit model to data.

[fitresult{7}, gof(7)] = fit( xData, yData, ft, opts );

% Plot fit with data.

figure( 'Name', 'Exponential Liquid P1' );

h = plot( fitresult{7}, xData, yData );

legend( h, 'yb_conc vs. time_post', 'Gamma 6 Particulate', 'Location', 'NorthEast' );

% Label axes

xlabel time_post

ylabel yb_conc

grid on

%% Fit: 'Gamma 3 Liquid P1'.

[xData, yData] = prepareCurveData( time_post_dose, co_concentration );

% Set up fittype and options.

ft = fittype( '0*(x<x0)+[a*[(d/(d-c))^3*exp(-c*(x-x0))-exp(-d*(x-x0))*((d/(d-c))^3+(d/(d-c))^2*d*(x-x0)+((d/(d-c))*d^2*(x-x0)^2)/2)]].*(x>=x0)', 'independent', 'x', 'dependent', 'y' );

opts = fitoptions( 'Method', 'NonlinearLeastSquares' );

opts.Display = 'Off';

opts.Lower = [-Inf 0 0 -Inf];

opts.MaxFunEvals = 1000000;

opts.StartPoint = [0.65375734866856 0.49417393663927 0.779051723231275 0.715037078400694];

opts.Upper = [Inf 1 1 Inf];

% Fit model to data.

[fitresult{8}, gof(8)] = fit( xData, yData, ft, opts );

% Plot fit with data.

figure( 'Name', 'Gamma 3 Liquid P1' );

h = plot( fitresult{8}, xData, yData );

legend( h, 'yb_conc vs. time_post', 'Gamma 6 Particulate', 'Location', 'NorthEast' );

% Label axes

xlabel time_post

ylabel yb_conc

grid on

%% Fit: 'Gamma 4 Liquid P1'.

[xData, yData] = prepareCurveData( time_post_dose, co_concentration );

% Set up fittype and options.

ft = fittype( '0*(x<x0)+[a*[(d/(d-c))^4*exp(-c*(x-x0))-exp(-d*(x-x0))*((d/(d-c))^4+(d/(d-c))^3*d*(x-x0)+((d/(d-c))^2*d^2*(x-x0)^2)/2+((d/(d-c))*d^3*(x-x0)^3)/6)]].*(x>=x0)', 'independent', 'x', 'dependent', 'y' );

opts = fitoptions( 'Method', 'NonlinearLeastSquares' );

opts.Display = 'Off';

opts.Lower = [-Inf 0 0 -Inf];

opts.MaxFunEvals = 10000;

opts.StartPoint = [0.903720560556316 0.890922504330789 0.334163052737496 0.698745832334794];

opts.Upper = [Inf 1 1 Inf];

% Fit model to data.

[fitresult{9}, gof(9)] = fit( xData, yData, ft, opts );

% Plot fit with data.

figure( 'Name', 'Gamma 4 Liquid P1' );

h = plot( fitresult{9}, xData, yData );

legend( h, 'yb_conc vs. time_post', 'Gamma 6 Particulate', 'Location', 'NorthEast' );

% Label axes

xlabel time_post

ylabel yb_conc

grid on

%% Fit: 'Gamma 5 Liquid P1'.

[xData, yData] = prepareCurveData( time_post_dose, co_concentration );

% Set up fittype and options.

ft = fittype( '0*(x<x0)+[a*[(d/(d-c))^5*exp(-c*(x-x0))-exp(-d*(x-x0))*((d/(d-c))^5+(d/(d-c))^4*d*(x-x0)+((d/(d-c))^3*d^2*(x-x0)^2)/2+((d/(d-c))^2*d^3*(x-x0)^3)/6+((d/(d-c))*d^4*(x-x0)^4)/24)]].*(x>=x0)', 'independent', 'x', 'dependent', 'y' );

opts = fitoptions( 'Method', 'NonlinearLeastSquares' );

opts.Display = 'Off';

opts.MaxFunEvals = 100000;

opts.MaxIter = 40000;

opts.Lower = [-Inf 0 0 -Inf];

opts.StartPoint = [0.197809826685929 0.0305409463046367 0.744074260367462 0.500022435590201];

opts.Upper = [Inf 1 1 Inf];

% Fit model to data.

[fitresult{10}, gof(10)] = fit( xData, yData, ft, opts );

% Plot fit with data.

figure( 'Name', 'Gamma 5 Liquid P1' );

h = plot( fitresult{10}, xData, yData );

legend( h, 'yb_conc vs. time_post', 'Gamma 6 Particulate', 'Location', 'NorthEast' );

% Label axes

xlabel time_post

ylabel yb_conc

grid on

%% Fit: 'Dhanoa Particulate P1'.

[xData, yData] = prepareCurveData( time_post_dose, yb_concentration );

% Set up fittype and options.

ft = fittype( 'a*exp(-k*x)*exp(-(N-2)*exp(-d*x))', 'independent', 'x', 'dependent', 'y' );

opts = fitoptions( 'Method', 'NonlinearLeastSquares' );

opts.Display = 'Off';

opts.Lower = [-Inf -Inf 0 0];

opts.MaxFunEvals = 6000;

opts.MaxIter = 4000;

opts.StartPoint = [0.62406008817369 0.679135540865748 0.395515215668593 0.367436648544477];

opts.Upper = [Inf Inf 1 1];

% Fit model to data.

[fitresult{11}, gof(11)] = fit( xData, yData, ft, opts );

% Plot fit with data.

figure( 'Name', 'Dhanoa Particulate P1' );

h = plot( fitresult{11}, xData, yData );

legend( h, 'yb_conc vs. time_post', 'Gamma 6 Particulate', 'Location', 'NorthEast' );

% Label axes

xlabel time_post

ylabel yb_conc

grid on

%% Fit: 'Dhanoa P1 Liquid'.

[xData, yData] = prepareCurveData( time_post_dose, co_concentration );

% Set up fittype and options.

ft = fittype( 'a*exp(-k*x)*exp(-(N-2)*exp(-d*x))', 'independent', 'x', 'dependent', 'y' );

opts = fitoptions( 'Method', 'NonlinearLeastSquares' );

opts.Display = 'Off';

opts.Lower = [-Inf -Inf 0 0];

opts.MaxFunEvals = 6000;

opts.MaxIter = 4000;

opts.StartPoint = [0.62406008817369 0.679135540865748 0.395515215668593 0.367436648544477];

opts.Upper = [Inf Inf 1 1];

% Fit model to data.

[fitresult{12}, gof(12)] = fit( xData, yData, ft, opts );

% Plot fit with data.

figure( 'Name', 'Dhanoa P1 Liquid' );

h = plot( fitresult{12}, xData, yData );

legend( h, 'yb_conc vs. time_post', 'Gamma 6 Particulate', 'Location', 'NorthEast' );

% Label axes

xlabel time_post

ylabel yb_conc

grid on

%% Fit: 'Gamma 6 Particulate'.

[xData, yData] = prepareCurveData( time_post, yb_conc );

% Set up fittype and options.

ft = fittype( '0*(x<x0)+[a*[(d/(d-c))^6*exp(-c*(x-x0))-exp(-d*(x-x0))*((d/(d-c))^6+(d/(d-c))^5*d*(x-x0)+((d/(d-c))^4*d^2*(x-x0)^2)/2+((d/(d-c))^3*d^3*(x-x0)^3)/6+((d/(d-c))^2*d^4*(x-x0)^4)/24+((d/(d-c))*d^5*(x-x0)^5)/120)]].*(x>=x0)', 'independent', 'x', 'dependent', 'y' );

opts = fitoptions( 'Method', 'NonlinearLeastSquares' );

opts.Display = 'Off';

opts.Lower = [-Inf 0 0 -Inf];

opts.StartPoint = [0.140360660621917 0.83446249179394 0.381558457093008 0.765516788149002];

opts.Upper = [Inf 1 1 Inf];

% Fit model to data.

[fitresult{13}, gof(13)] = fit( xData, yData, ft, opts );

% Plot fit with data.

figure( 'Name', 'Gamma 6 Particulate' );

h = plot( fitresult{13}, xData, yData );

legend( h, 'yb_conc vs. time_post', 'Gamma 6 Particulate', 'Location', 'NorthEast' );

% Label axes

xlabel time_post

ylabel yb_conc

grid on

%% Fit: 'Gamma 6 Liquid'.

[xData, yData] = prepareCurveData( time_post, co_conc );

% Set up fittype and options.

ft = fittype( '0*(x<x0)+[a*[(d/(d-c))^6*exp(-c*(x-x0))-exp(-d*(x-x0))*((d/(d-c))^6+(d/(d-c))^5*d*(x-x0)+((d/(d-c))^4*d^2*(x-x0)^2)/2+((d/(d-c))^3*d^3*(x-x0)^3)/6+((d/(d-c))^2*d^4*(x-x0)^4)/24+((d/(d-c))*d^5*(x-x0)^5)/120)]].*(x>=x0)', 'independent', 'x', 'dependent', 'y' );

opts = fitoptions( 'Method', 'NonlinearLeastSquares' );

opts.Display = 'Off';

opts.Lower = [-Inf 0 0 -Inf];

opts.StartPoint = [0.445586200710899 0.646313010111265 0.709364830858073 0.754686681982361];

opts.Upper = [Inf 1 1 Inf];

% Fit model to data.

[fitresult{14}, gof(14)] = fit( xData, yData, ft, opts );

% Plot fit with data.

figure( 'Name', 'Gamma 6 Liquid' );

h = plot( fitresult{14}, xData, yData );

legend( h, 'yb_conc vs. time_post', 'Gamma 6 Particulate', 'Location', 'NorthEast' );

% Label axes

xlabel time_post

ylabel yb_conc

grid on
