# Supplementary material for: Digestibility and Retention Time of Coastal Bermudagrass (Cynodon dactylon) Hay by Horses
Source: Animals (Basel). 2019 Dec 14;9(12):1148. doi: 10.3390/ani9121148 (PMC6940996; doi:10.3390/ani9121148)
Supplement: Supplementary file 1 [file animals-09-01148-s001.zip › Supplementary Figures - Individual Horse Marker Excretion Within Diet.pdf]

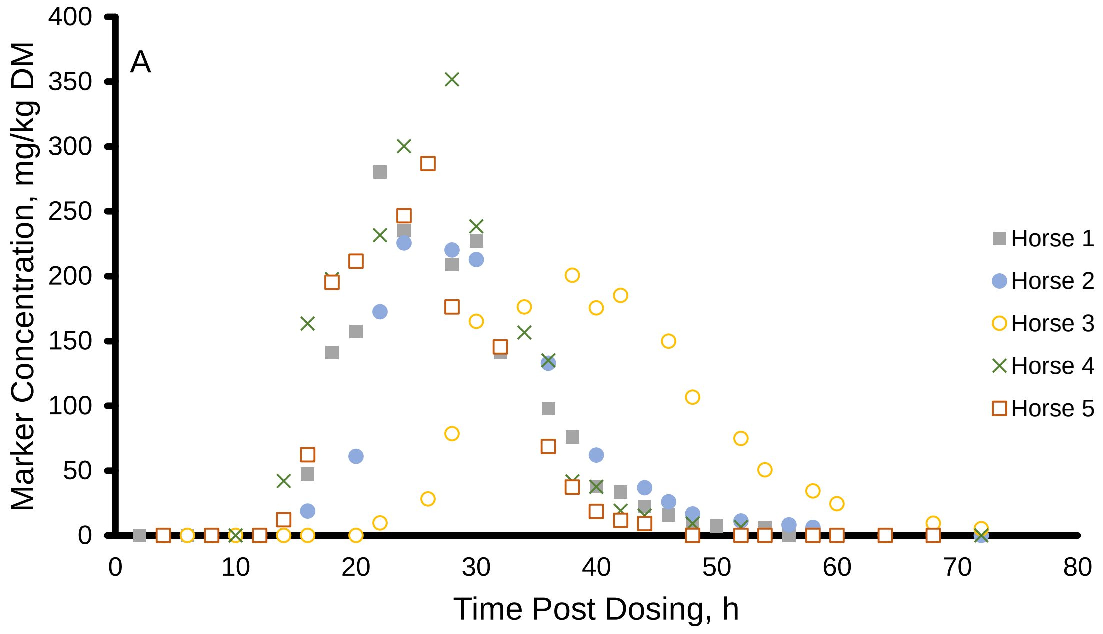

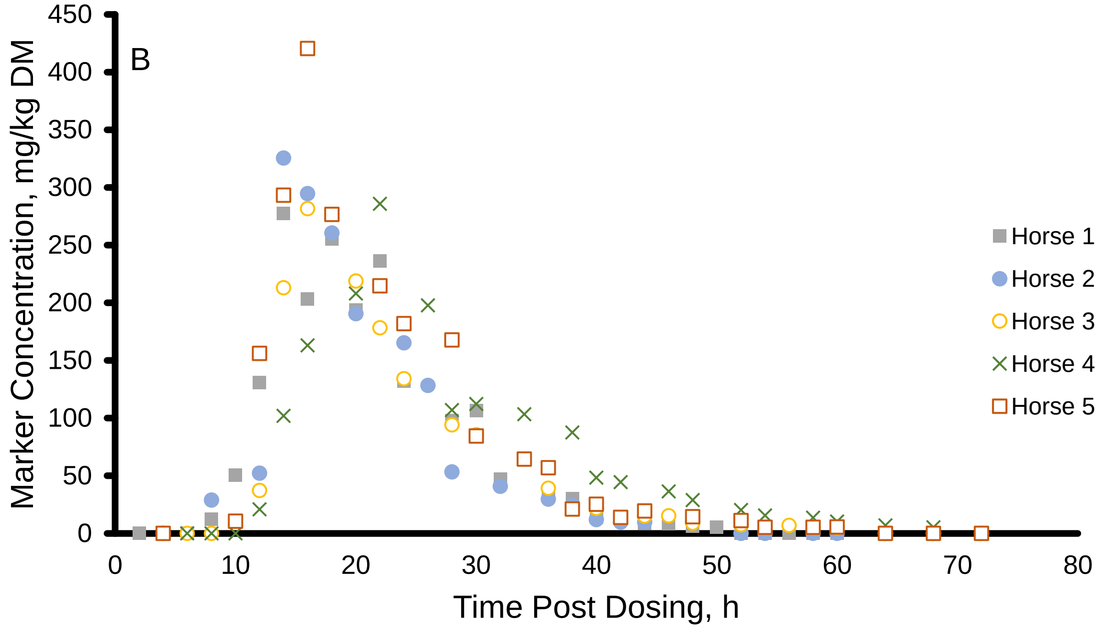

Supplemental Figure 1. *Fecal marker excretion of (a) Yb and (b) Co after dosing external markers when horses were fed alfalfa hay.*

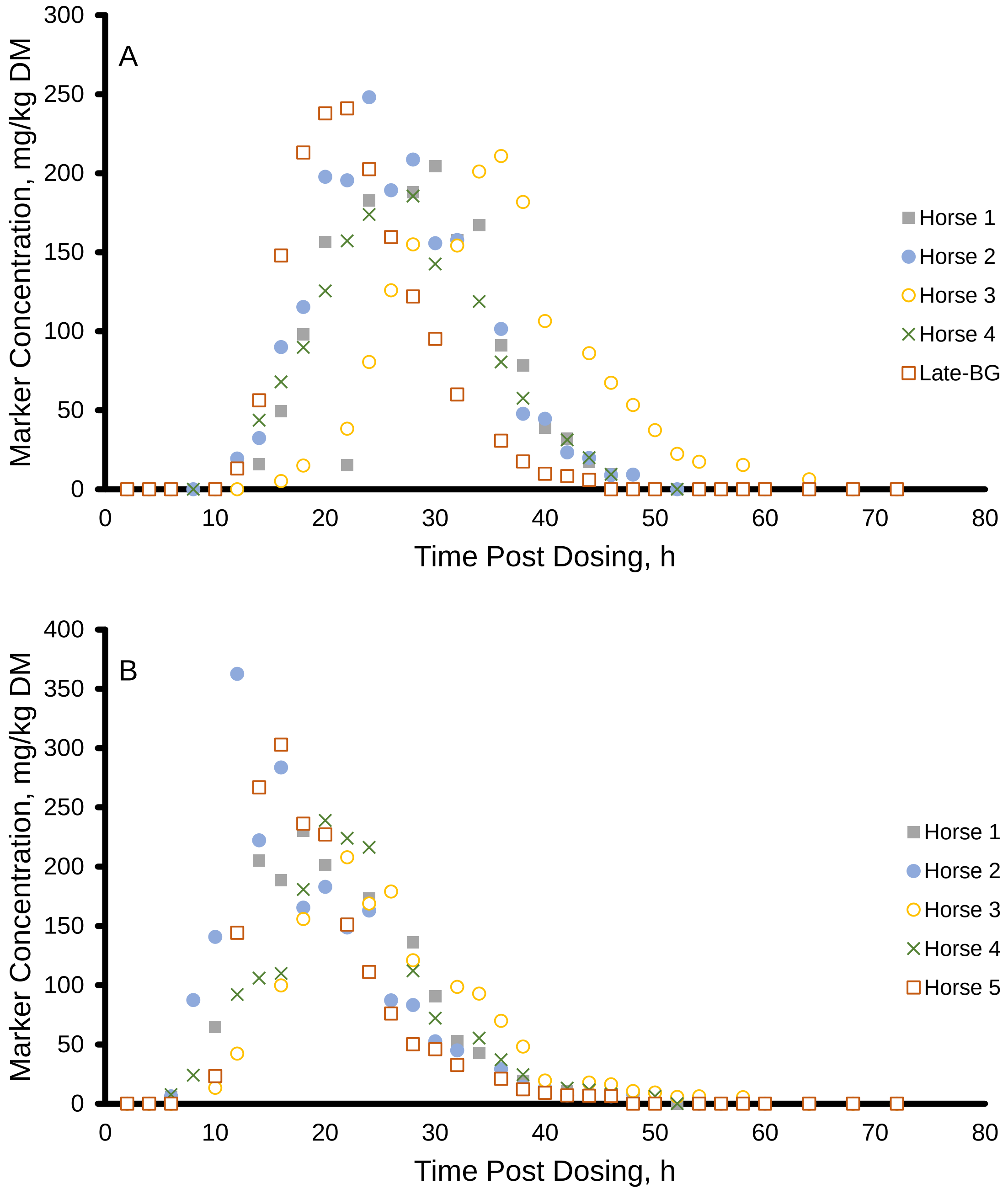

Supplemental Figure 2. *Fecal marker excretion of (a) Yb and (b) Co after dosing external markers when horses were fed orchardgrass hay.*

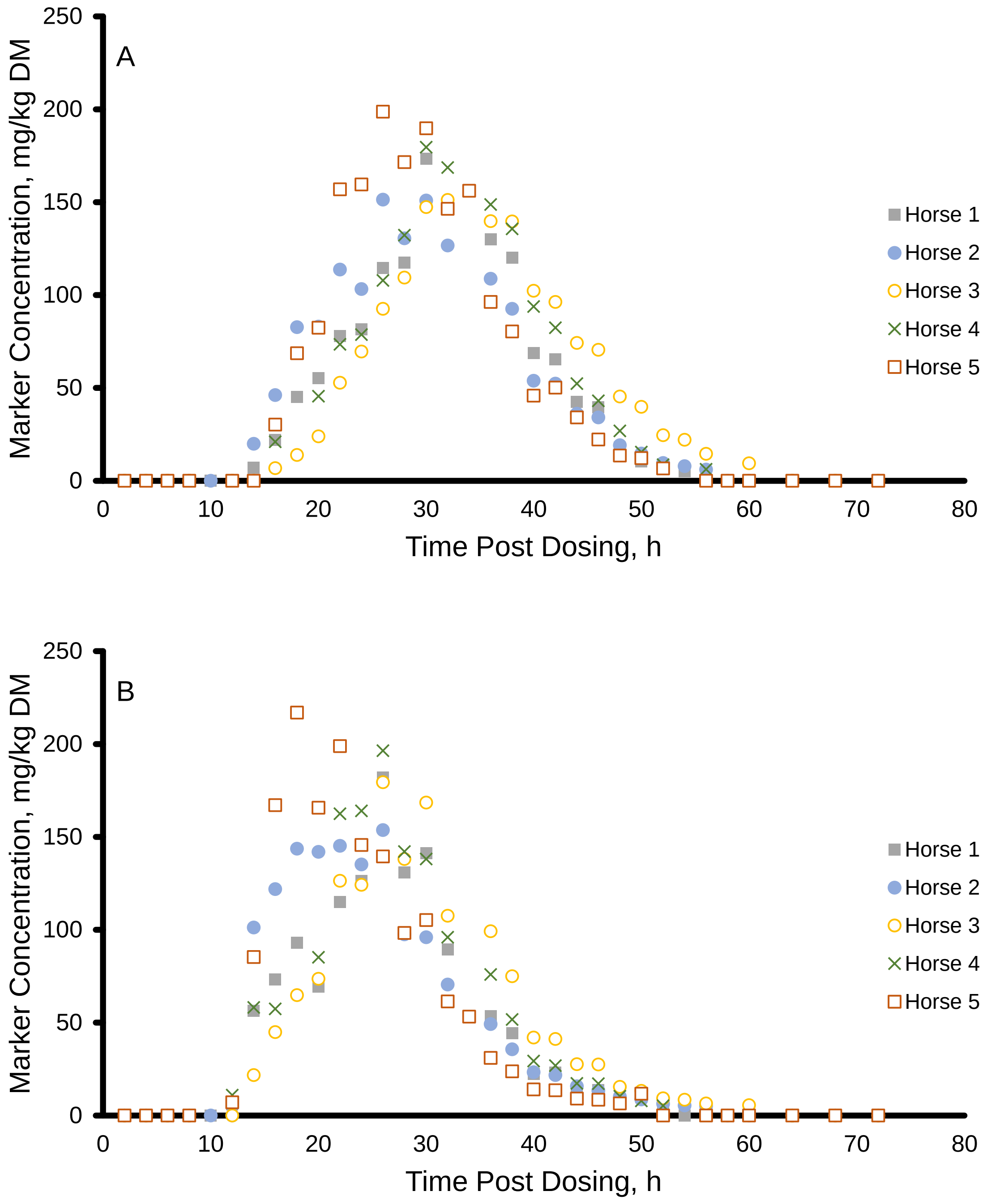

Supplemental Figure 3. *Fecal marker excretion of (a) Yb and (b) Co after dosing external markers when horses were fed Coastal bermudagrass hay harvested at 4-wk of regrowth.*

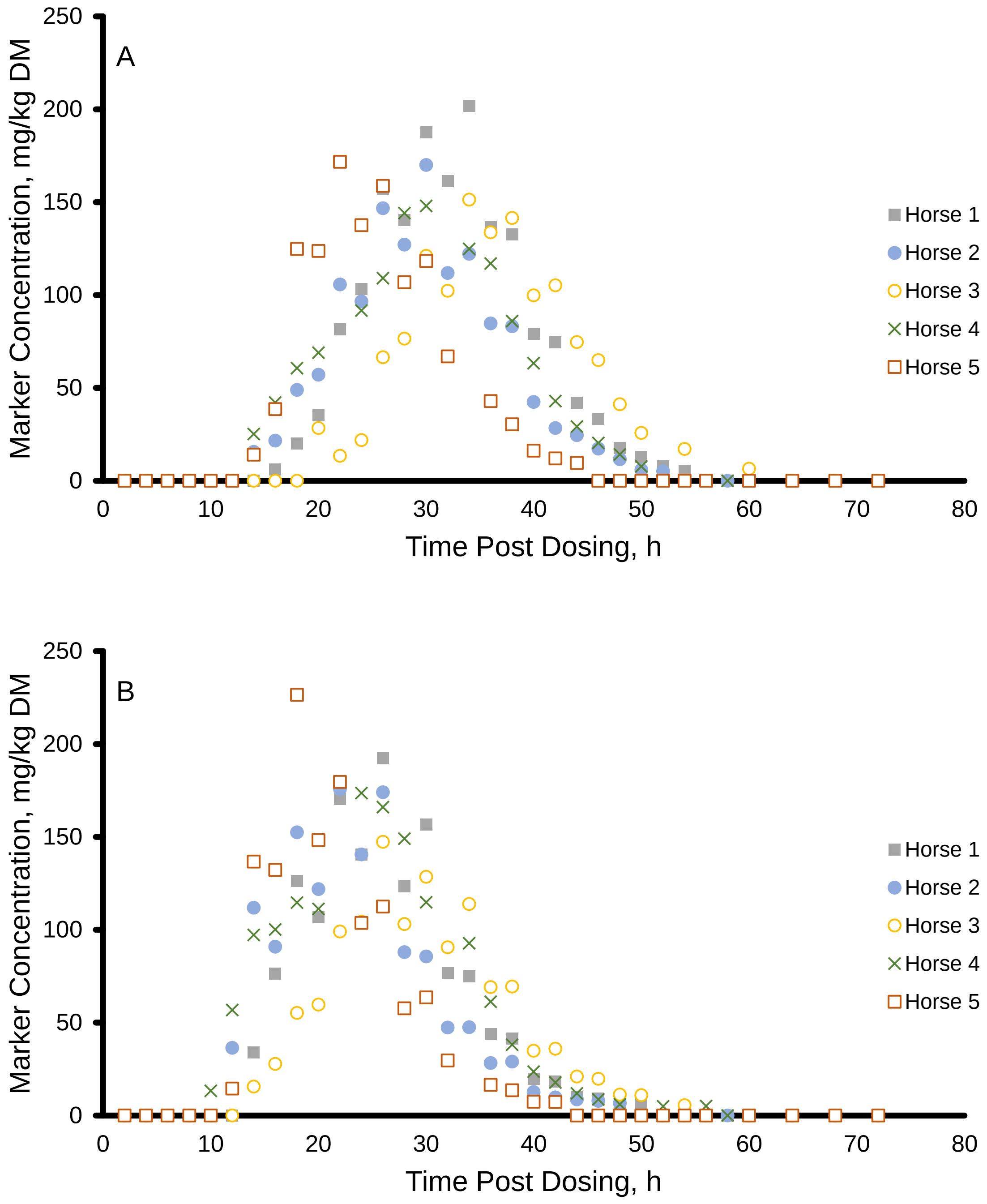

Supplemental Figure 4. *Fecal marker excretion of (a) Yb and (b) Co after dosing external markers when horses were fed Coastal bermudagrass hay harvested at 8-wk of regrowth.*

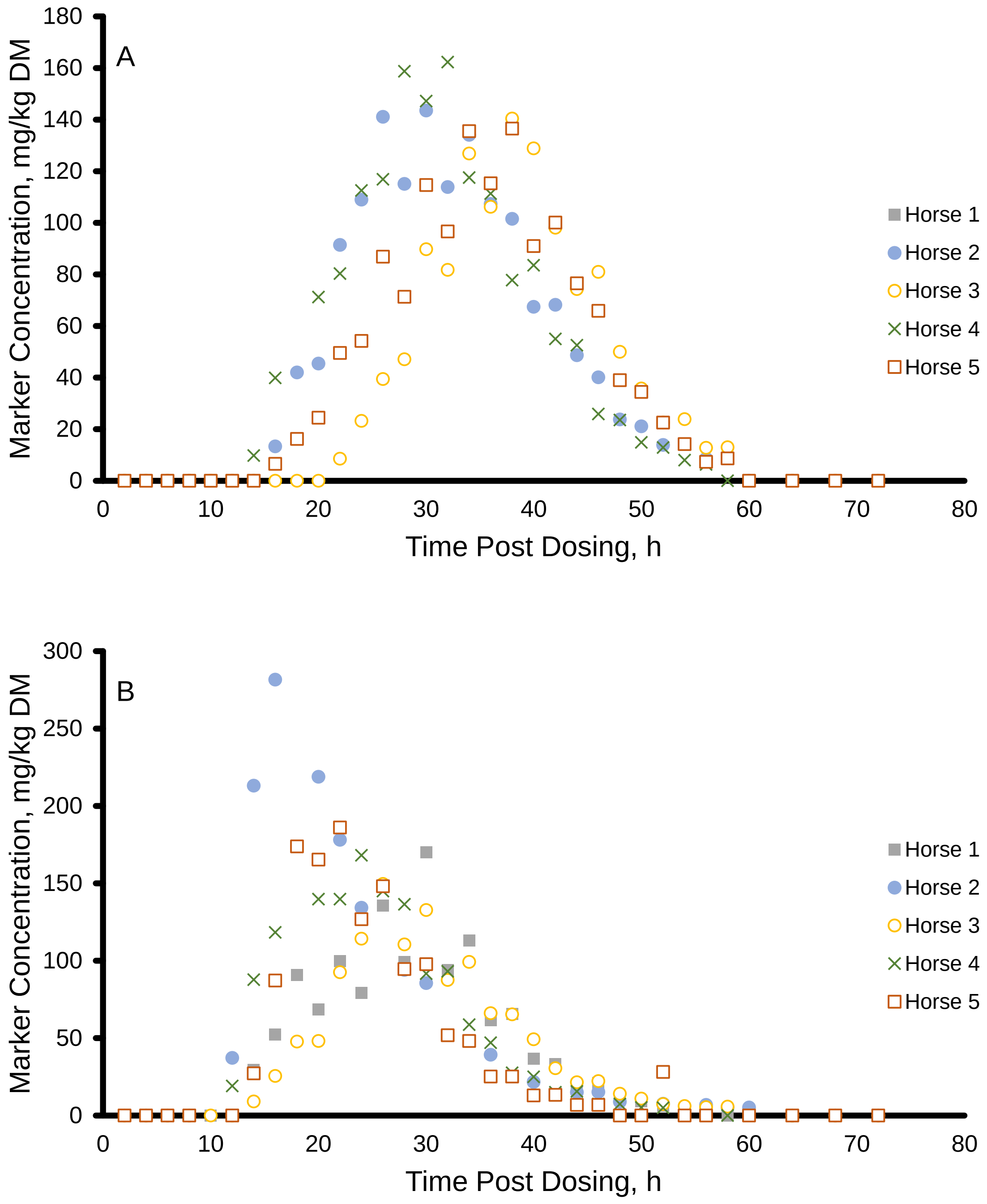

Supplemental Figure 5. *Fecal marker excretion of (a) Yb and (b) Co after dosing external markers when horses were fed Coastal bermudagrass hay harvested at 8-wk of regrowth.*
